# Clinical Practice Guidelines Adherence and the Impact on Health Outcomes in Oncology: A Systematic Review and Meta‐Analysis

**DOI:** 10.1002/gin2.70030

**Published:** 2025-07-21

**Authors:** Nofisat Ismaila, Lawrence Mbuagbaw, Jinhui Ma, Lehana Thabane

**Affiliations:** ^1^ Department of Health Research Methods, Evidence, and Impact McMaster University Hamilton Ontario Canada; ^2^ American Society of Clinical Oncology Alexandria Virginia USA; ^3^ Department of Anesthesia McMaster University Hamilton Ontario Canada; ^4^ Department of Pediatrics McMaster University Hamilton Ontario Canada; ^5^ Biostatistics Unit, Father Sean O'Sullivan Research Centre, St Joseph's Healthcare Hamilton Ontario Canada; ^6^ Centre for Development of Best Practices in Health (CDBPH), Yaoundé Central Hospital Yaoundé Cameroon; ^7^ Division of Epidemiology and Biostatistics, Department of Global Health Stellenbosch University Cape Town South Africa; ^8^ St. Joseph's Healthcare‐Hamilton Hamilton Ontario Canada; ^9^ Faculty of Health Sciences University of Johannesburg Johannesburg South Africa

**Keywords:** guideline adherence, meta‐analysis, neoplasms, overall survival, systematic review

## Abstract

**Aim:**

Clinical practice guidelines (CPGs) are developed to standardize care by providing physicians and decision‐makers with evidence‐based recommendations. This study analyzed the effects of guideline adherence on clinical outcomes in patients with cancer.

**Methods:**

Five electronic databases were searched through December 2024. Inclusion criteria were randomized control trials (RCT), prospective and retrospective cohort studies on patients with cancer managed with CPG adherent vs. non‐adherent care. Two reviewers screened studies for inclusion, extracted data and assessed the quality of studies. Outcomes of interest included overall survival (OS), disease‐free survival (DFS), and quality of life. A random‐effects model was used to pool the results. Certainty of evidence (CoE) was assessed using the GRADE approach.

**Results:**

Seventy‐five studies were included. Results suggest that adherence to CPG was associated with improved OS in all disease sites (hazard ratio [HR] = 0.64 (95% confidence interval [CI]: 0.59 to 0.70); *p* < 0.0001, *I* ^2^: 97%; low CoE). Subgroup analysis by disease site showed similar results except for gynecological cancers where adherence showed no statistically significant improvement in OS (HR = 0.76 (95% CI: 0.53 to 1.08); *p* = 0.13, *I* ^2^: 77%; low CoE). Further analysis of studies in breast cancer with 5 years follow‐up average showed improvement in OS (HR = 0.70 (95% CI: 0.64 to 0.77); *p* < 0.001, *I* ^2^: 0%; low CoE).

**Conclusion:**

Evidence suggests that clinician's adherence to CPG recommendations may improve survival outcomes in patients with cancer. However, the evidence is largely based on retrospective observational studies, highlighting need for prospective trials to further elucidate these associations and better guide clinical practice.

## Summary

- What Is Already Known on the Subject
  ∘ Adherence to clinical practice guidelines (CPGs)(CPGs) is expected to improve patient outcomes and some primary studies in oncology support this association.
  ∘ However, other studies report no significant difference in patient outcome regardless of whether clinicians adhere to CPG recommendations or not.
  ∘ A systematic synthesis of evidence to clarify this association is necessary to inform guideline developers and researchers on whether CPGs achieve their intended impact.
- What This Study Adds
  ∘ This systematic literature review and meta‐analysis provides a comprehensive evaluation of the impact of CPG adherence in oncology.
  ∘ The findings highlight cancer disease sites where evidence is limited and the need for more prospective studies to strengthen the evidence base.
- Implications for Practice, Research, and Policy
  ∘ Given the significant time and resources required to develop CPGs, it is essential to assess whether they achieve their intended impact.
  ∘ While adherence to CPGs appears to be beneficial for improving clinical outcome in patients with cancer, further research is needed to strengthen the evidence base and enhance guideline implementation.
  ∘ Ensuring that CPGs translate into meaningful patient benefits and efficient resource utilization should remain a key focus.

## 1 Introduction

Clinical practice guidelines (CPGs) are developed to standardize care by providing physicians and decision‐makers with evidence‐based recommendations that are based on the systematic review of available evidence [1]. There are currently about 3000 CPGs available in different languages in the Guidelines International Network library database [2]. About 30% of these CPGs are oncology guidelines and most are developed by reputable guideline development organizations such as the American Society of Clinical Oncology, the National Comprehensive Cancer Network (NCCN), and the European Society of Medical Oncology in North America and Europe. According to the US National Library of Science, CPG adherence means conformity in fulfilling or following official, recognized, or institutional requirements, recommendations, protocols, pathways, or other standards [3, 4]. Adherence to these CPGs is expected to result in better patient outcomes [5, 6, 7]. However, since most CPGs are based on synthesis of evidence from clinical trials, which are usually performed in a controlled setting with experienced investigators and select patient populations, there is the likelihood of overestimating the benefits of these interventions and underestimating the harms.

Furthermore, non‐adherence to CPGs has been reported in some studies and reasons identified included lack of resources or infrastructure, variation in techniques in surgical interventions, and variation in hospital protocols or procedures [8, 9, 10]. There is also the issue of rapidly changing guidance based on the development of new drugs in cancer care that would require clinicians to be up to date in the management of their patients. Other factors that could play a role in CPGs nonadherence could include clinical reasons like contraindications or patient preferences for treatments [11].

Developing high‐quality evidence‐based CPGs can be time‐consuming and costly. Thus, it is imperative to know that these resources do not go to waste or to what extent they are providing the intended impact. Hence, this study evaluates the impact of CPG adherence on health outcomes in patients with cancer using published clinical practice data.

## 2 Methods

This systematic review is reported according to the Preferred Reporting Items for Systematic Reviews and Meta‐analysis (PRISMA) statement [12]. The protocol for this review was registered in PROSPERO (CRD42021285492).

### 2.1 Patient and Public Involvement

This study did not include patient and public involvement in the selection of the topic, methods used, outcomes accessed or dissemination plans.

### 2.2 Literature Search Strategy

We searched MEDLINE, EMBASE, PsychINFO, CINAHL, and the Cochrane Controlled Trials Register through December 2024 with the use of a comprehensive and exhaustive search strategy developed by a health science librarian with expertise in systematic review literature searching. The details of the search strategy are available in Appendix SA.

### 2.3 Eligibility Criteria

Studies involving adult cancer patients being managed in healthcare centers were included. Interventions of interest were adherence to CPGs. The comparator was non‐adherence to CPGs (i.e., usual care without use of adherence to guideline). The outcomes of interest included patient‐related outcomes (overall survival (OS), disease‐free survival (DFS), recurrence‐free survival (RFS), incidence‐based mortality, harm, and quality of life (QOL). We included systematic reviews (to identify primary studies), randomized controlled trials, and observational studies (cohort, before and after, and case‐control). We excluded articles that were non‐English articles, abstracts, conference proceedings, and studies involving guideline use for preventative or investigative procedures such as imaging or laboratory screening.

### 2.4 Data Screening Process

The bibliographic records from each database were uploaded into DistillerSR software [13]. All duplicate articles were removed by running the automated search in DistillerSR. The title and abstract screening were done by one reviewer (N.I.) and audited by a second reviewer (R.G.). A full‐text screening of relevant studies identified from the first level of screening was done in duplicate by two reviewers (N.I. and R.G). Disagreements were resolved by discussion or with the help of a third reviewer.

### 2.5 Data Extraction and Risk of Bias Assessment

We extracted data on the characteristics of the included studies (e.g., last name of first author, publication year, country, study design, patient population, disease site, number of patients, intervention setting [primary, tertiary, inpatient, outpatient], guideline used, characteristic of intervention and control arms and study aim) and results on the outcomes of interest. For studies with multiple publications or similar source data, we included the publication with the longest follow up and with more complete data to avoid double counting populations or events.

The risk of bias assessment was conducted by using specific tools that are relevant to the study designs including the Cochrane risk of bias (RoB) 2 tool to assess randomized controlled trials (RCTs) [14] and the Newcastle‐Ottawa Scale (NOS) for observational studies [15]. The NOS scale was used to grade studies according to a score of 0 (highest risk of bias) to 9 (lowest risk of bias). Cut‐off points of < 5 (high risk of bias); 5–7 (moderate risk of bias); > 7 (low risk of bias) was applied. Data extraction and risk of bias assessment were conducted independently by two reviewers (N.I. and B.H.).

### 2.6 Data Analysis

Descriptive statistics, including means, frequencies and percentages, were used to summarize the main characteristics of the included studies. When appropriate, results from comparable groups of studies were combined using a random effects meta‐analysis using the Review Manager software from the Cochrane Collaboration [16]. For survival outcomes (OS, RFS and DFS) comparing guideline adherent and non‐adherent groups, only studies reporting hazard ratios (HR) were included in the meta‐analysis. Pooled survival results are expressed as HR with 95% confidence intervals (CI) or presented narratively when statistical pooling was not feasible. HRs < 1 denotes survival advantage for guideline adherence and HRs > 1 denotes advantage for guideline non‐adherence. Adjusted HRs were preferred over unadjusted estimates to minimize confounding and better reflect the independent association between adherence to clinical practice guidelines and patient outcomes. The adjustments made by the original studies commonly included covariates such as age, sex, disease stage, comorbidities, ethnicity, smoking status, socioeconomic status, and treatment modality. Statistical heterogeneity was assessed using the Cochrane Q test (with a significance threshold set at *p* < 0.10), *I*² index, and *τ*² tests, with the restricted maximum likelihood method used to estimate the between‐study variance [17, 18]. In cases of high statistical heterogeneity (*I* ^2^ > 75%), subgroup analysis were performed on the pooled estimates, stratified by disease type and study duration to explore potential sources of heterogeneity. To provide an estimate of the expected range of effect sizes in future studies, a 95% prediction interval (PI) was calculated. The prediction interval incorporates both the uncertainty in the summary effect estimate and the variability across studies. The certainty of evidence (CoE) was evaluated by applying the Grading of Recommendation Assessment, Development and Evaluation (GRADE) approach [19]. A minimally contextualized approach was used to assess the certainty and the null effect was set as the threshold. A funnel plot was visually inspected to assess potential publication bias or small‐study effects.

To assess the influence of individual studies on the overall meta‐analytic results, a leave‐one‐out sensitivity analysis was conducted. In this approach, each study was sequentially removed, and the meta‐analysis was re‐run to observe the change in the pooled effect size, confidence intervals and heterogeneity. Further analysis was also conducted by study design by excluding the pre‐post and prospective cohort studies to assess their impact on the pooled effect size.

## 3 Results

**Figure 1:**
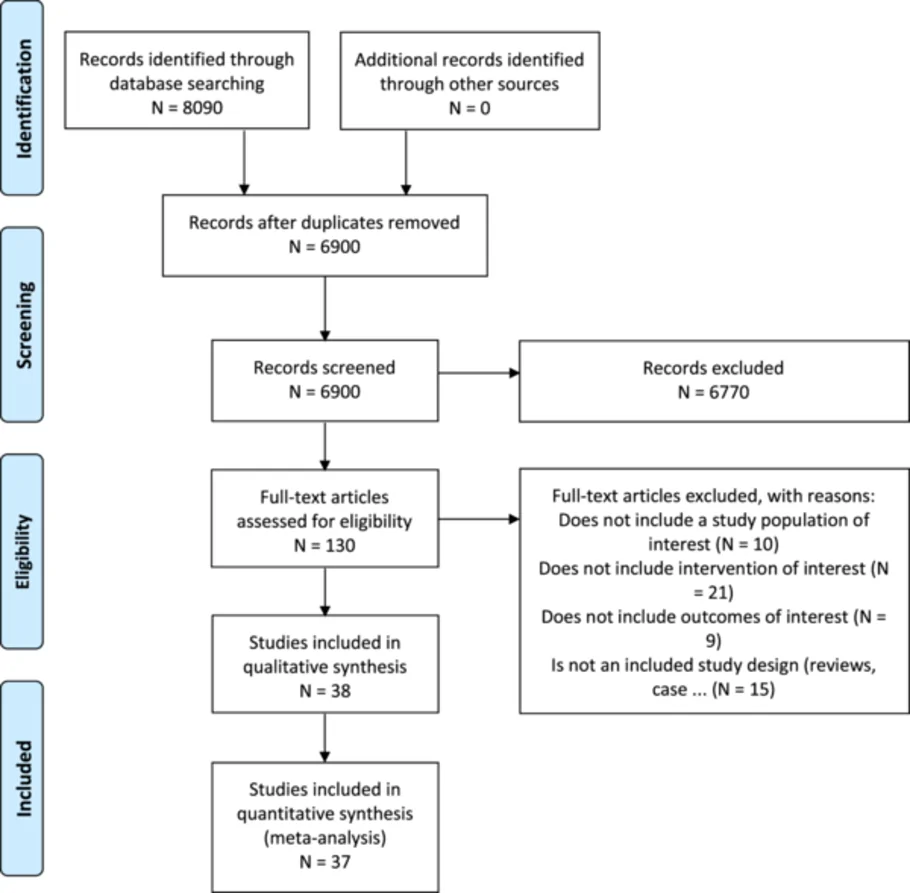
PRISMA flow diagram of literature search.

The literature search identified 6900 unique citations after duplicates were removed. After the title and abstract and full‐text screening, a total of 75 citations were eligible for inclusion in this review [6, 8, 9, 10, 20, 21, 22, 23, 24, 25, 26, 27, 28, 29, 30, 31, 32, 33, 34, 35, 36, 37, 38, 39, 40, 41, 42, 43, 44, 45, 46, 47, 48, 49, 50, 51, 52, 53, 54, 55, 56, 57, 58, 59, 60, 61, 62, 63, 64, 65, 66, 67, 68, 69, 70, 71, 72, 73, 74, 75, 76, 77, 78, 79, 80, 81, 82, 83, 84, 85, 86, 87, 88, 89, 90, 91]. Details on the levels of screening and reasons for exclusion of articles can be found in the PRISMA flow diagram (Figure 1) and Supporting Information.

### 3.1 Summary of the Included Studies

Most of the studies included patients with gastrointestinal (*n* = 24, 32%) and breast cancers (*n* = 17, 23%) and were conducted mostly in Europe (*n* = 38, 51%) and North America (*n* = 26, 35%). Eighty‐eight percent of the studies were retrospective cohort studies [6, 8, 9, 10, 20, 21, 22, 23, 24, 25, 26, 27, 28, 29, 30, 31, 32, 33, 34, 35, 36, 37, 38, 39, 40, 41, 42, 43, 44, 45, 46, 47, 48, 49, 50, 51, 52, 53, 54, 55, 56, 57, 58, 59, 60, 61, 62, 63, 64, 65, 66, 67, 68, 69, 70, 71, 72, 83, 84, 85, 86, 87, 88, 89, 90, 91]. Five studies used a pre‐post study design to evaluate patient outcomes before and after the introduction of CPGs [73, 74, 75, 76, 77]. Three prospective cohort studies evaluated the impact of CPG adherence on overall survival in patients with prostate [78], hepatocellular carcinoma [79], and pancreatic cancer [80]. One multi‐center RCT focused on an adjuvant protocol for pancreatic adenocarcinoma to evaluate the impact of adherence to specified radiotherapy protocol on survival and toxicity [81]. The primary outcome reported in these studies included OS, DFS, and RFS while all studies reported data on the CPG adherence rate. There were no studies identified evaluating the impact on QOL. Thirty‐two percent of the studies used the NCCN guidelines while 15% used the German National Consensus S3 Guidelines. The European Association of Urology Guidelines was about 7% while the other guidelines used ranged between 1% and 4% of the studies. A summary of the characteristics of studies included are in Table 1. Further details on individual studies are available in the Supporting Information.

**Table 1:** Characteristics of included studies (*n* = 75).

Study characteristics	*n* (%)
Study population (cancer disease site)	
Gastrointestinal	24 (32.0%)
Breast	17 (22.7%)
Gynecological	11 (14.7%)
Genitourinary	8 (10.7%)
Head and neck	9 (12.0%)
Thoracic	5 (6.7%)
Melanoma	1 (1.3%)
Location	
Asia	7 (9.3%)
Australia	4 (5.3%)
Europe	38 (50.7%)
North America	26 (34.7%)
Study design	
Randomized control trial	1 (1.3%)
Prospective cohort	3 (4.0%)
Retrospective cohort	66 (88.0%)
Pre‐Post	5 (6.7%)
Guidelines used^a^	
National comprehensive cancer network	24 (32.0%)
German national consensus S3 guideline	11 (14.7%)
European association of urology	5 (6.7%)
European society for medical oncology	3 (4.0%)
American thyroid association guidelines	3 (4.0%)
Comprehensive cancer center middle netherlands	3 (4.0%)
Barcelona clinic liver cancer guideline	3 (4.0%)
Australian cancer council guidelines	2 (2.7%)
American college of surgeons	2 (2.7%)
Japan society of gynecologic oncology	2 (2.7%)
National dutch guideline	2 (2.7%)
Standardized treatment protocol	2 (2.7%)
American urological association	1 (1.3%)
Catalonia colorectal cancer guidelines	1 (1.3%)
French association of urology guideline	1 (1.3%)
French national health authority	1 (1.3%)
Integrated healthcare process for cancer (Granada) and Onco‐Guide (Girona)	1 (1.3%)
International federation of gynecology and obstetrics	1 (1.3%)
Malaysian clinical practice guidelines	1 (1.3%)
National collaborating centre for cancer, cardiff	1 (1.3%)
International society of geriatric oncology	1 (1.3%)
Scottish intercollegiate guidelines network	1 (1.3%)
European society of gynecologic oncology	1 (1.3%)
Italian association of medical oncology	1 (1.3%)
Asian pacific association for the study of the liver; european association for the study of the liver; american association for the study of liver diseases	1 (1.3%)
Not specified	7 (9.3%)

^a^ Some studies used more than one guideline.

### 3.2 Description of Studies by Disease Sites

#### 3.2.1 Gastrointestinal

The gastrointestinal disease site was represented by 1 RCT, 2 prospective cohort, and 21 retrospective studies (Table 1). These include six studies in Stage II and III colon cancers [20, 24, 25, 26, 27, 30], four studies on hepatocellular cancers [33, 34, 79, 89], five studies on rectal cancers [31, 32, 35, 84, 88], three on pancreatic cancers [22, 80, 81]; two studies each on colorectal cancers [23, 28] and gastric cancer [29, 83]; one study each on cholangiocarcinoma [85] and biliary duct cancer [21]. Half of the studies were conducted in Europe, while the other half was in North America. Study follow‐up ranged between 2 and 10 years. Most of the studies reported significant survival benefit in patients whose management adhered to CPG treatment recommendations except for two out of the four studies in hepatocellular carcinoma [33, 79] and one study in Stage III colon cancer [24] reporting nonsignificant findings.

#### 3.2.2 Breast Cancer

There were 17 retrospective cohort studies identified in this disease group. Thirteen studies included patients with primary breast cancer [10, 37, 40, 41, 42, 44, 45, 46, 47, 48, 49, 50, 75], two studies with triple‐negative breast cancer [38, 92], and two with metastatic breast cancer [39, 93]. About 90% of the studies in this disease group were conducted in Europe and their study duration ranged from 2 to 15 years. A similar outcome of OS benefit from adhering to CPG recommendations was reported in about 80% of the studies [10, 37, 38, 40, 41, 42, 44, 45, 46, 47, 48, 49, 50, 75], three studies however reported nonsignificant differences in survival rates between adherence to CPG recommendations vs. non‐adherence [36, 39, 43].

#### 3.2.3 Gynecologic

Another disease site that had a good number of studies included was the gynecologic cancer disease site. There were three retrospective studies each on cervical [54, 55, 59], and endometrial cancer [58, 73, 74], four studies on ovarian cancer [9, 56, 57, 90] and one study that included patients with HIV that had a mix of all these cancers [60]. Half of these studies reported statistical significant differences on CPG adherence on OS [9, 54, 55, 57, 60, 90], while the other half reported no difference in survival outcomes [56, 58, 59, 73, 74]. Sample sizes ranged from 57 to 3237 with an average follow‐up of 48 months. Two of the studies on endometrial cancers applied a pre‐post study design to evaluate the impact of CPG adherence on OS. Both showed similar results as one evaluated an updated CPG recommendation which showed an uptake in the use of the recommended intervention but no significant difference in RFS or OS between pre‐ to post‐CPG exposure [74] while the other also did not find a significant difference in pre‐ or post‐CPG exposure on OS (HR = 0.891; *p* = 0.160) [73].

#### 3.2.4 Genitourinary

Patients with genitourinary cancers were included in five retrospective, one prospective cohort, and two pre‐post studies. These included four studies on bladder cancer [8, 53, 76, 86], two studies on penile [51, 77] and one study each on testicular [52] and prostate cancer [78]. Most studies were done in Europe with a median follow‐up of ranging between 9 months and 5 years. Study findings were favorable to CPG adherence except for the prospective study on prostate cancer which showed no significant difference in OS at 24 with probability (95% CI) of 0.39 (0.31–0.48) vs. 0.47 (0.38–0.55); logrank *p* = 0.8) and 36‐months (0.33 (0.25–0.42) vs. 0.41 (0.33–0.50); logrank *p* = 0.8) [78].

#### 3.2.5 Other Disease Sites

The remaining 15 retrospective cohort studies comprised of patients with head and neck [61, 62, 63, 64, 65, 66, 67, 68, 87], thoracic [6, 69, 70, 71, 91] and melanoma [72] cancers and they all reported significant OS benefit from adhering to CPG recommendations except for two studies in head and neck cancer and one study in thoracic cancer. One of the head and neck cancer study comprised of 159 patients ≥ 80 years of age with a median follow‐up of 391 days. Though treatment was in accordance with NCCN recommendations in 59% of patients and deviation in 41%, there was no difference in 2‐year OS (62% vs. 66%, *p* = 0.50) [63]. The second study was a retrospective cohort study using medical records in 217 patients with stage II and III tongue and floor of mouth squamous carcinoma. Most of the treatment adhered to the CPG recommendations (55%) however there was no statistically significant difference in tumor recurrence between adherence or non‐adherence for stage II (*p* = 0.68) or stage III disease (*p* = 0.97) [65]. The only thoracic cancer study that did not show any difference in survival based on adherence involved 2020 patients with node negative NSCLC. Adherence rate to NCCN guideline was about 51% and with a median follow up of 18.7 months, HR was 1.00 (*p* = 0.992) [71].

### 3.3 Adverse Events

Impact of CPG adherence on toxicity was reported in a secondary analysis of an RCT on adjuvant chemotherapy and chemoradiotherapy in patients with resected pancreatic cancers which involved the use of specified radiotherapy protocol guidelines [81]. While there was no statistically significant difference between radiotherapy adherent and non‐adherent arms, there was a trend toward increased toxicity for the non‐adherent patients for both hematologic Grade 4 (*p* = 0.08) and non‐hematologic toxicity (*p* = 0.06) in patients receiving gemcitabine.

Impact of CPG adherence on postsurgical complications were also discussed in three studies. Verbeek et al reported that adherence to the American Thyroid Association guidelines resulted in fewer cancer‐related reoperations (mean = 0.24 vs. 0.60; *p* = 0.027) and more biochemical cure (40.9% vs. 20%; *p* = 0.038) in patients with medullary thyroid cancers [64]. van Vuuren et al on the other hand reported 66% of octogenarians with rectal cancer who were treated according to guidelines experienced complications vs. 34% of those treated otherwise (*p* = 0.02) [32]. Lastly, Foster et al evaluated adverse outcomes in non‐adherence to melanoma guidelines and reported postoperative complications in 17% of patients. These complications were 3.4‐fold higher for patients treated in a margin‐noncompliant fashion and 2.4‐fold higher for patients treated in a lymph‐node‐noncompliant manner (*p* < 0.001 for both) [72].

None of the studies reported impact of CPG adherence on incidence‐based mortality or quality of life.

### 3.4 Study Quality Assessment

The NOS was used to assess the 74 cohort studies. The assessment showed 35% of these studies had a low risk of bias and 65% had a moderate risk of bias with no study rated as a high risk of bias. The most common sources of risk of bias were related to the selection of the nonexposed cohort, outcome assessment, and adequacy of follow‐up. The RoB 2 tool was used to assess the single RCT included, and the assessment indicated a low risk of bias.

### 3.5 Guideline Adherence

The average CPG adherence rates by disease sites ranged between 56% and 85%. One of the studies involving the use of a breast cancer CPG reported the highest average overall CPG adherence (97%) which was defined as the number of patients who received the recommended therapy for treatment [37]. This was followed by a study in gastrointestinal (96%) [80] and gynecological cancers (90%) [59]. The lowest adherence rates were reported in studies in genitourinary (23%) [8], gynecological (26.7%) [9], and breast cancers (31%) [10].

### 3.6 Effect of Guideline Adherence on Overall Survival

**Figure 2:**
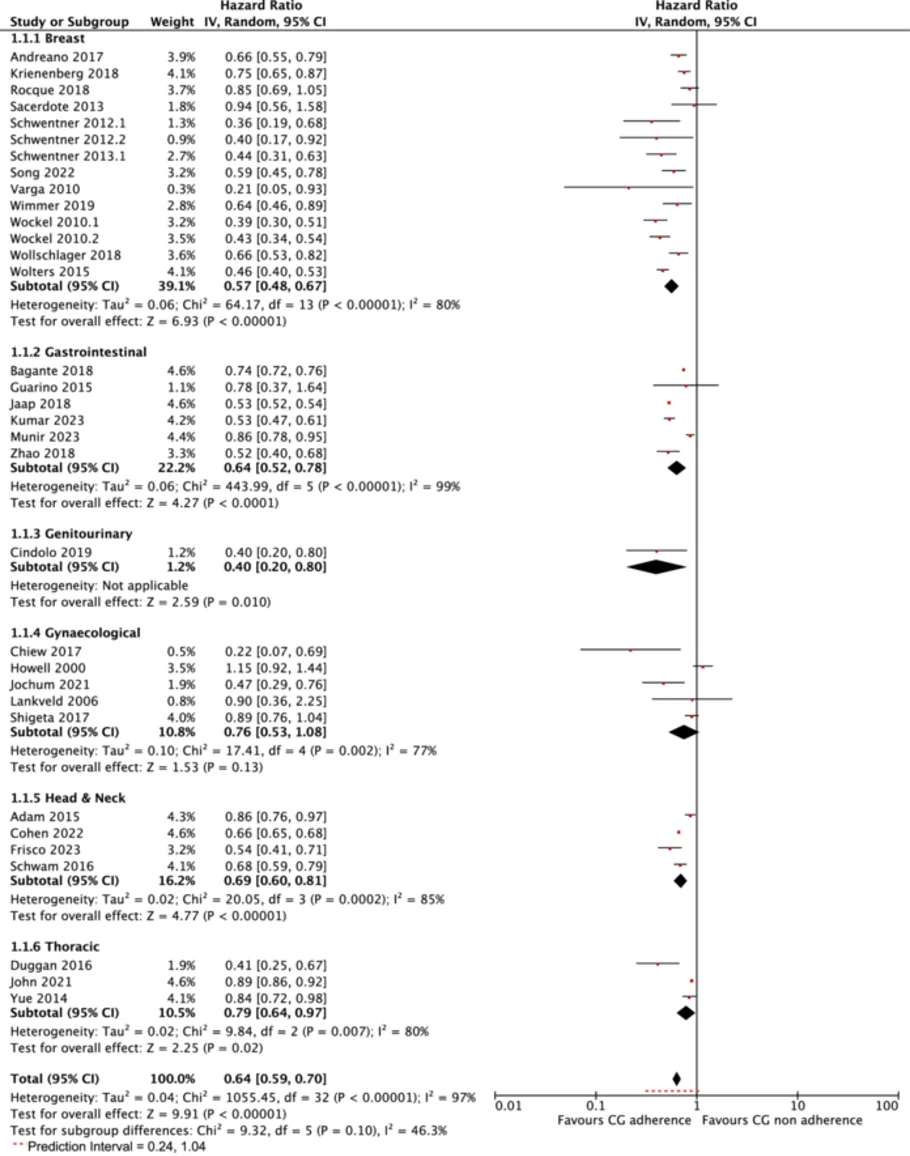
Random effects meta‐analysis showing association between guideline adherence and overall survival in all disease sites.

Thirty‐three studies comprising of 30 retrospective, two pre‐post and one prospective studies out of the 75 studies reported data on OS with similar outcome measures (Table 2) [6, 10, 21, 22, 25, 37, 38, 39, 40, 41, 42, 44, 45, 46, 47, 50, 51, 55, 57, 58, 59, 61, 62, 66, 67, 69, 70, 73, 75, 79, 82, 84, 85]. Adherence to guidelines may improve OS based on the result from a random effects meta‐analysis (HR = 0.64; 95% CI: 0.59 to 0.70; *p* < 0.0001, *I* ^2^: 97%; low certainty of evidence(CoE)). The CoE was rated down because data was pooled from 33 observational studies encompassing diverse disease populations and varying follow up periods (Table 3 and Figure 2). The analysis revealed high statistical heterogeneity (*I* ^2^ = 97%, *p* < 0.0001) and test for subgroup differences revealed moderate heterogeneity between subgroups (*I* ^2^ = 46.3%), but the difference in effect sizes across subgroups was not statistically significant (*p* = 0.10), indicating that the grouping variable does not strongly explain the observed variation in outcomes. Furthermore, the 95% prediction interval, which accounts for both the uncertainty in the pooled effect size and the between‐study heterogeneity, ranged from 0.24 to 1.04. This wide interval suggests considerable variability in effect sizes across individual studies and highlights the potential for differing results in future studies conducted under similar conditions (Figure 2).

**Table 2:** Characteristics of studies included in meta‐analysis.

Author last name and year	Institution(*N*)/Registry database	Guideline used	Outcomes	Adherence Rate	Risk of bias
Study country	Patient population	Total *N*	Follow‐up duration (mean or total * )	HR (95% CI, *p‐*value)	NOS score
**Gastrointestinal (*n* = 7)**					
*Prospective cohort Study*					
Guarino, 2015 Europe	Institution (1) Hepatocellular carcinoma	Barcelona Clinic Liver Cancer guidelines *N* = 92	Adherence, survival 3 years	73% OS: 1.29 (0.62 to 2.71, NR)	Moderate 6
*Retrospective cohort studies*					
Munir 2023 North America	Registry cholangiocarcinoma	NCCN *N* = 4519	Adherence, survival 5 years	42% OS: 0.86 (0.78 to 0.95, *p* < 0.05)	Low 8
Kumar 2023 North America	Registry Anal cancer	NCCN *N* = 4740	Adherence, survival 5 years	81% OS: 1.87 (1.66 to 2.12, *p* < 0.0001)	Low 8
van den Berg 2021 Europe	Institution (7) Stage III colon cancer	Not specified *N* = 575	Adherence, survival 47 months	61% RFS: 0.42 (0.29 to 0.62, *p* < 0.001)	Low 7
Jaap, 2018 North America	National Cancer Database Stage I/II pancreatic cancer	NCCN *N* = 52450	Adherence, survival 8 years*	37% OS: 0.53 (0.52 to 0.54, *p* < 0.0001)	Moderate 6
Bagante, 2018 North America	National Cancer Database Biliary tract cancers	NCCN Comprehensive Cancer Center Middle Netherlands (CCCMN) *N* = 176536	Adherence, survival 10 years*	42% OS: 0.74 (0.72 to 0.76, *p* < 0.001)	Moderate 6
Zhao, 2018 North America	Texas Cancer Registry/Medicare Stage II and III colon cancer patients ≥ 66 years	NCCN *N* = 6028	Adherence, survival 5 years*	69% OS: 1.94 (1.50 to 2.50, *p* < 0.01)^a^	Moderate 7
**Breast (*n* = 14)**					
*Pre‐Post studies*					
Kreienberg 2018 Europe	Institution (17) patients with primary breast cancer	German national consensus S3 guideline *N* = 8323	Adherence, survival 5 years	57% OS: 0.75 (0.65 to 0.85, *p* < 0.001) RFS: 0.79 (0.68 to 0.92, *p* = 0.003)	Low 7
Sacerdote 2013 Europe	Institution (1) Patients with incident breast cancer	Piedmont guidelines *N* = 1092	Adherence, quality of care, mortality 2 years*	72% OS: 0.94 (0.56 to 1.58, NR)	Moderate 7
*Retrospective cohort studies*					
Song, 2022 Asia	Registry (2) Universiti Malaya Breast Cancer Registry, Ramsay Sime Darby Breast Cancer Registry Women with stage I‐III breast cancer	Malaysian Clinical Practice Guidelines for Management of Breast Cancer Second Edition *N* = 3100	Adherence, survival 5 years	63% OS: 1.69 (1.29 to 2.22, *p* < 0.01)^b^	Moderate 7
Wimmer 2019 Europe	Tumor Centre Regensburg Registry Patients with invasive breast cancer after breast‐conserving therapy (BCT)	German national consensus S3 guideline *N* = 6370	Adherence, survival 6.3 years	97% OS: 0.64 (0.46 to 0.89, *p* = 0.007) RFS: 0.2 (0.16 to 0.25, *p* < 0.001)	Low 8
Wollschlager, 2018 Europe	Institution (17) BRENDA DATABASE Comorbid breast cancer patients	German national consensus S3 guideline *N* = 2137	Adherence, survival 5.7 years	NR OS: 1.51 (1.19 to 1.90, *p* < 0.001) DFS: 1.71 (1.41 to 2.07, *p* < 0.0001)	Low 9
Rocque, 2018 North America	SEER‐Medicare database Women with de novo stage IV metastatic breast cancer	NCCN *N* = 988	Adherence, survival Costs to Medicare/healthcare utilization 2.1 years	81% OS: 0.85 (0.69 to 1.05, NR)	Moderate 7
Andreano, 2017 Europe	Lomdardy, Italy Cancer Registry database Women with an epithelial breast cancer	National Collaborating Centre for Cancer, Cardiff and ESMO *N* = 6333	Adherence, survival 5.6 years	69% OS: 0.66 (0.55 to 0.79, NR)	Low 8
Wolters, 2015 Europe	Institution (17) Women with breast cancer	Not specified *N* = 9061	Adherence, survival 10 years*	52% OS: 0.46 (0.40 to 0.53, *p* < 0.001) RFS: 0.51 (0.44 to 0.59, *p* < 0.001)	Moderate 6
Schwentner, 2013.1 Europe	Institution (17) BRENDA DATABASE histologically confirmed invasive breast cancer	German national consensus S3 guideline *N* = 9433	Adherence, survival 15 years*	NR OS: 2.25 (1.60 to 3.18, *p* < 0.001)^c^ RFS: 1.91 (1.44 to 2.53, *p* < 0.001)^c^	Moderate 6
Schwentner, 2012.1 Europe	Institution (17) BRENDA DATABASE Histologically confirmed invasive breast cancer	German national consensus S3 guideline *N* = 5292	Adherence, survival 10 years*	31% OS: 2.80 (1.46 to 5.36, *p* = 0.002)^d^ RFS: 3.49 (1.89 to 6.41, *p* < 0.001)^d^	Moderate 7
Schwentner, 2012.2 Europe	Institution (17) BRENDA DATABASE Histologically confirmed invasive breast cancer with TNBC	German national consensus S3 guideline *N* = 3658	Adherence, survival 5 years	33.% OS: 2.51 (1.08 to 5.81, *p* = 0.032) RFS: 1.98 (1.11 to 3.55, *p* = 0.021)	Low 8
Wockel, 2010.1 Europe	Institution (17) BRENDA DATABASE Histologically confirmed invasive breast cancer	German national consensus S3 guideline *N* = 3976	Adherence, survival 5 years	52% OS: 2.57 (1.96 to 3.37, *p* = 0.0001) RFS: 2.20 (1.74 to 2.79, *p* = 0.0001)	Moderate 6
Wockel, 2010.2 Europe	Institution (17) BRENDA DATABASE histologically confirmed invasive breast cancer	German national consensus S3 guideline *N *= 2231	Adherence, survival 13 years	70% OS: 2.32 (1.84 to 2.92, *p* < 0.001) RFS: 1.72 (1.38 to 2.15, *p* < 0.001)	Moderate 6
Varga, 2010 Europe	Institution (17) BRENDA DATABASE Breast cancer patients (the very young under 35 years and the younger patients between 36 and 55, all premenopausal)	German national consensus S3 guideline *N* = 1778	Adherence, survival mean follow‐up was 59.6 months for patients diagnosed at < 35 years and 62.1 months for patients diagnosed at 35–55 years.	54% OS: 4.73 (1.08 to 20.69, *p* = 0.039) RFS: 2.95 (1.11 to 7.83, *p* = 0.030)	Low 8
**Gynecological (*n* = 6)**					
*Pre‐Post study*					
Shigeta, 2017 Asia	Japan Society of Obstetrics and Gynecology (JSOG) cancer registration system Endometrial cancer	JSGO *N* = 65,241	Adherence, survival 12 years*	NR OS: 0.89 (0.76 to 1.04, *p* = 0.160)	Moderate 6
*Retrospective cohort studies*					
Jochum, 2021 Europe	Institution (12) Invasive epithelial ovarian cancer	ESMO‐European Society of Gynecologic Oncology (ESGO) ovarian cancer guideline *N* = 1463	Adherence, survival 30 months	30% OS: 2.14 (1.32 to 3.47, *p* < 0.01)	Low 8
Chiew, 2017 Australia	Southwest Sydney Local Health District and Sydney Local Health District Clinical Cancer Registry Cervical cancer	NCCN, JSGO, ESMO, NICE *N* = 208	Adherence, survival 5 years	54% OS: 0.22 (0.07 to 0.69, *p* = 0.015)^e^	Moderate 6
Lee, 2015 Asia	Seoul National University Hospital registry database ovarian cancer	NCCN *N* = 266	Adherence, survival 8.4 years	27% RFS: 0.36 (0.15 to 0.88, NR) DFS: 0.42 (0.16 to 1.10, NR)	Low 7
Lankveld, 2006 Europe	Regional Cancer Registry Netherlands Early‐stage endometrial cancer	Comprehensive Cancer Center Middle Netherlands *N* = 359	Adherence, survival 68.8 months	87% OS: 0.9 (0.36 to 2.25, NR)	Low 8
Howell, 2000 North America	SEER database Cervical cancer	International Federation of Gynecology and Obstetrics *N* = 5952	Adherence, survival 7 years*	90% OS: 0.87 (0.70 to 1.09, NR)	Moderate 6
**Genitourinary (*n* = 3)**					
*Pre‐Post studies*					
Ulvskog 2023 Europe	Registry Penile cancer	Swedish guideline *N* = 274	Adherence, survival 5 years	45% DFS: 0.41 (0.28 to 0.61, NR)	Low 8
Sorenby. 2019 Europe	Institution (1) non‐muscle invasive bladder cancer	Standardized treatment protocol *N* = 275	Adherence, survival 36 months	78.5% RFS: 0.65 (0.43 to 1.0, *p* = 0.05)	Low 7
*Retrospective cohort study*					
Cindolo, 2019 Europe	Institution Penile cancer	European Association of Urology *N* = 425	Adherence, survival 17 months	74% OS: 0.40 (0.20 to 0.80, *p* = 0.014)	Moderate 7
**Head & neck (*n* = 4)**					
*Retrospective cohort studies*					
Frisco 2023 North America	SEER Data Base Thyroid cancer	ATA *N* = 2898	Adherence, survival 5 years	68% OS: 1.85 (1.42 to 2.43, *p* < 0.001)^f^	Low 8
Cohen 2022 North America	National Cancer Data Base stage III and IVA HNC	NCCN *N* = 100,074	Adherence, survival 5 years	74% OS: 1.51 (1.48 to 1.54, *p* < 0.001)	Low 8
Schwam, 2016 North America	National Cancer Data Base Nasopharyngeal Carcinoma	NCCN *N* = 1741	Adherence, survival 46.8 months	74% OS: 1.46 (1.25 to 1.69, NR)	Moderate 7
Adam, 2015 North America	National Cancer Data Base Thyroid cancer	NCCN American Thyroid Association *N* = 39687	Adherence, survival 75 months	94% OS: 1.16 (1.03 to 1.31, *p* = 0.02)	Moderate 7
**Thoracic (*n* = 3)**					
*Retrospective cohort studies*					
John, 2021 North America	Registry Database Advanced NSCLC	NCCN *N* = 28784	Adherence, survival 11.7 months	69% OS: 0.89 (0.86 to 0.92, *p* < 0.01)	Moderate 7
Duggan, 2016 Australia	SW Sydney Clinical Cancer Registry Stage I‐IIIb NSCLC	Australian Cancer Council guidelines *N* = 592	Adherence, survival 54 months	66% OS: 0.41 (0.25 to 0.67, *p* < 0.001)^g^	Moderate 7
Yue, 2014 Asia	Institution (1) NSCLC	NCCN American College of Surgeons Oncology Group, OSI Pharmaceutical RADIANT trial and IASLC *N* = 2711	Adherence, survival 96 months*	60% OS: 0.84 (0.72 to 0.98, *p* = 0.032)	Low 8

Abbreviations: ATA, American Thyroid Association; BRENDA, breast cancer care under evidence‐based guidelines; DFS, disease free survival; ESMO, European Society of Medical Oncology; HR, hazard rate; MBC, Metastatic breast cancer; N, number; NCCN, National Comprehensive Cancer Network; NICE, National Institute for Health and Care Excellence; NSCLC, Non‐Small Cell Lung Cancer; OS, Overall survival; RFS, Recurrence free survival; SEER, Surveillance, Epidemiology, and End Results.

In cases where multiple HRs were reported in the studies, the following are the ones selected for extraction based on their relevance to the eligibility criteria:

^a^ HR reported for non‐adherent surgery vs. surgery adherence in patients with stage II colon cancer.

^b^ HR for chemotherapy group.

^c^ HR for Participant‐non conform vs. Participant‐conform groups.

^d^ HR for chemotherapy in bilateral breast cancer patients.

^e^ HR for Stage I and II cervical cancer.

^f^ HR for localized disease group.

^g^ HR is for Stage I NSCLC group.

* Total follow up duration reported in study.

**Table 3:** GRADE certainty of evidence.

Adherence to CPG compared to non‐adherence to CPG for patients with cancer						
Patient or population: patients with cancer						
Intervention: adherence to CPG						
Comparison: non‐adherence to CPG						
	Anticipated absolute effects* (95% CI)					
Outcomes	Risk with non‐adherence to CPG	Risk with adherence to CPG	Relative effect (95% CI)	№ of participants (studies)	Certainty of the evidence (GRADE)	Comments
OS—All disease sites follow‐up: Range 1.3 years to 15 years	144 per 1000^a^	95 per 1000 (88 to 103)	HR 0.64 (0.0.59 to 0.70)	447597 (33 observational studies)^b^	⊕⊕◯◯ Low^c^	Adherence to CPG may result in an improvement in the OS in all disease sites but the evidence is uncertain.
OS—Breast subgroup follow‐up: Median 5 years	267 per 1000	195 per 1000 (180 to 212)	HR 0.70 (0.64 to 0.77)	17964 (5 observational studies)	⊕⊕◯◯ Low	The evidence suggests adherence to CPG may improve OS in breast cancer disease subgroup.

*The risk in the intervention group (and its 95% confidence interval) is based on the assumed risk in the comparison group and the relative effect of the intervention (and its 95% CI).

Abbreviations: CI: confidence interval; HR: hazard Ratio.

GRADE Working Group grades of evidence.

High certainty: we are very confident that the true effect lies close to that of the estimate of the effect. Moderate certainty: we are moderately confident in the effect estimate: the true effect is likely to be close to the estimate of the effect, but there is a possibility that it is substantially different.

Low certainty: our confidence in the effect estimate is limited: the true effect may be substantially different from the estimate of the effect.

Very low certainty: we have very little confidence in the effect estimate: the true effect is likely to be substantially different from the estimate of effect.

^a^ Only 19 out of the 33 studies either reported data on event rate or data to extrapolate event rate that was used in the calculation of anticipated absolute effects.

^b^ HR reported is for the 33 studies that were pooled but the total *N* of participants reported here is for the 19 studies with data on event rate.

^c^ This meta‐analysis pooled data from 33 observational studies encompassing diverse disease populations and varying follow up periods. The analysis revealed high statistical heterogeneity (*p* < 0.0001; *I* ^2^ = 97%) that could not be adequately accounted for through subgroup analysis.

**Figure 3:**
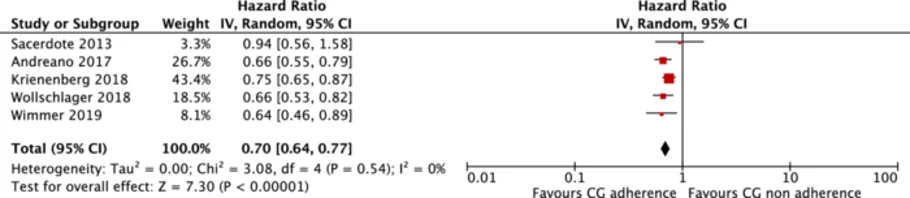
Random effects meta‐analysis showing association between adherence to breast cancer guidelines and overall survival rate.

Additional subgroup analysis was conducted using studies involving patients with a homogenous disease stage and a follow‐up duration of up to 5 years. This was feasible only within the breast cancer subgroup, which had the most studies with data available for pooling (*n* = 14). However, only five of these studies met the criteria for inclusion in this subgroup analysis [37, 40, 41, 44, 75]. The pooled results showed that adherence to guidelines may improve OS in this more homogenous patient population HR = 0.70 (95% CI: 0.64 to 0.77); *p* < 0.001, *I* ^2^: 0%; GRADE: low certainty of evidence) (Figure 3 and Table 3).

The leave‐one‐out sensitivity analysis performed showed that the exclusion of individual studies did not substantially alter the pooled effect size or reduce heterogeneity in the overall analysis, with *I* ^2^ values consistently ranging between 95% and 97%. This indicates that the high heterogeneity observed is not driven by any single study. However, within the disease subgroups, two studies notably influenced the pooled effect size and *I* ^2^ within their respective groups. In the head and neck cancer subgroup, the exclusion of Adam et al. [62] resulted in a minor change in the pooled effect size (from 0.66 to 0.69) but substantially reduced *I* ^2^ from 85% to 14%. Similarly, in the thoracic cancer subgroup, the exclusion of Duggan et al. [70] increased the pooled effect size from 0.79 to 0.89 and reduced *I* ^2^ from 80% to 0%. These results, detailed in Supporting Information Table S2 and Figure S6, suggest that the observed heterogeneity within these subgroups may be partially attributed to the influence of these individual studies.

Further subgroup analysis by study design done by excluding the pre‐post and prospective cohort studies, and grouping by study duration (Supporting Information Figures S4 and S5), did not significantly alter the pooled effect size or heterogeneity, indicating that the overall findings were robust.

#### 3.6.1 Recurrence and Disease‐Free Survival

Twelve studies reported data on RFS [9, 10, 30, 37, 38, 42, 46, 47, 49, 75, 76, 82] and three studies on DFS (Table 2) [9, 44, 77]. Both analyses showed similar findings as the OS analysis of all studies (HR = 0.45 (95% CI: 0.35 to 0.59); *p* < 0.0001, *I* ^2^: 90%; low CoE and HR = 0.51 (95% CI: 0.39 to 0.66); *p* < 0.0001, *I* ^2^: 32%; low CoE) for RFS and DFS respectively (Supporting Information S1: Figures S1 and S2).

#### 3.6.2 Assessment of Publication Bias

A funnel plot was created to visually assess the presence of publication bias or small‐study effects. The plot appeared symmetric, suggesting that publication bias is unlikely to have influenced the results of this meta‐analysis (Supporting Information S1: Figure S3).

**Figure 4:**
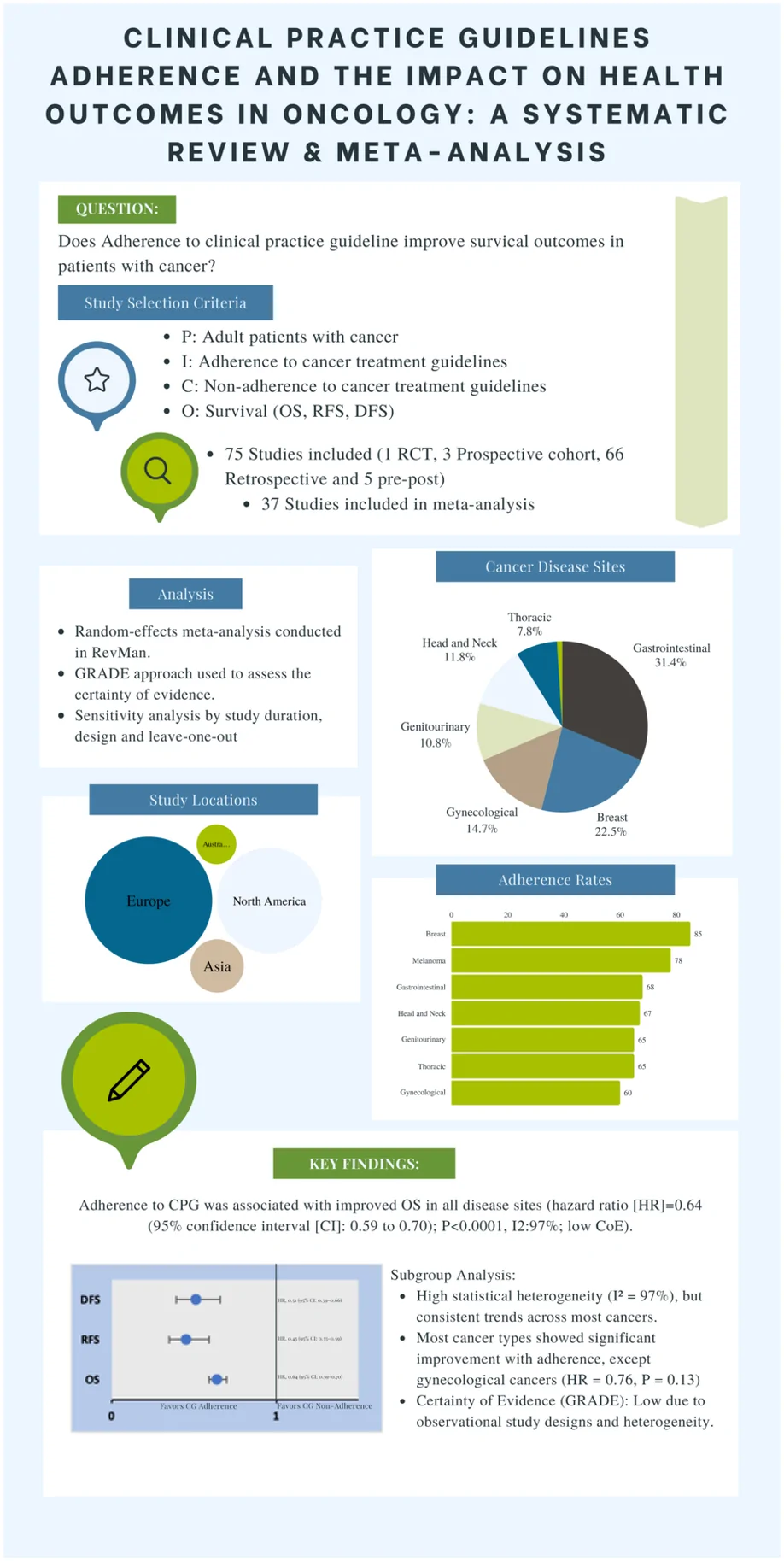
Infographic showing key findings from the systematic review and meta‐analysis.

A visual summary of key findings is presented in the infographic (Figure 4).

## 4 Discussion

In this study, we sought to evaluate the impact of adherence to CPGs on patient outcomes in oncology with the use of a systematic review of evidence and meta‐analysis of data where possible. Our analysis showed that adherence to CPGs may improve OS in all disease sites, but the evidence is very uncertain. Most of the other individual studies that could not be pooled also reported some improvement in OS, DFS, and RFS.

The findings in the breast cancer disease site are similar to a systematic review and meta‐analysis of 4 studies done by Ricci‐Cabello et al 2020 [5]. However, this study was limited to observational studies done only in European Union countries which limits generalization of the study findings. Another systematic review on adherence to cervical cancer treatment CPG also provided a narrative report of their findings due to a lack of homogenous studies on this topic [7].

In addition, the average rate of guideline adherence reported in these studies was about 60%. Some of the reasons for non‐adherence or lack of compliance to guideline recommendations were poorly reported in some studies while some studies reported a lack of documentation in the hospital records. This suboptimal level of adherence to guidelines has been reported in other systematic reviews [94, 95] and all of these could affect the estimation of the magnitude of the impact of CPG adherence on patient outcomes.

### 4.1 Strengths and Limitations

We followed a rigorous methodology according to the Cochrane Handbook of Systematic Reviews and the PRISMA checklist for reporting systematic reviews. A thorough literature search was conducted with the use of 5 electronic databases. Data extraction was detailed, and specific tools were used to evaluate the risk of bias based on study design. We also ensured that the evidence assessment was completed using GRADE to assess the certainty of evidence.

However, this study had some limitations. First, the available studies that were pooled were all retrospective registry study designs which are inherently subject to reporting and selection bias and issues with incomplete data. Second, none of the studies gathered any quality‐of‐life data on these patients or included any qualitative reporting on patients' or clinicians' views on guideline adherence. This could have helped get some perspective on what could be contributing to the low adherence rate in some studies.

Some other issues to note is that several of the meta‐analyses showed very high *I* ^2^ ranging from 78% to 99%. Despite further subgroup and sensitivity analysis, the *I* ^2^ was still high in most instances. Some of the likely sources of heterogeneity could be the clinical (different staging within the disease sites), variations in guidelines, sample sizes, and duration of study. The reliance on a small number of studies in certain subgroup analyses, particularly those with unequal weights is also a limitation. Specifically, the subgroup analysis for the breast cancer disease site with 5 years follow up duration was based on only five studies, which limits the robustness and generalizability of the findings. Additionally, the unequal weights across these studies may have influenced the overall effect estimate, as studies with larger sample sizes or more precise estimates exert greater influence on the pooled results. While the results of the subgroup analysis are statistically significant with high heterogeneity, the small number of studies and unequal weighting warrant caution in interpreting these findings.

Furthermore, despite the overall high heterogeneity observed in the meta‐analysis (*I*² = 97%), subgroup analyses did not fully account for all potential sources of this variation. This may be due to the variability in study design, patient population, or other unmeasured factors.

Also, about half of the studies included used either the NCCN or the German consensus guidelines. It is important to note that these guidelines are developed from consensus amongst experts rather than based on a systematic review of evidence which would offer the strongest level of evidence to support any association between adherence to these guideline recommendations and patient outcomes.

Lastly, the prediction interval (0.24 to 1.04) illustrates substantial variability in the effect sizes of included studies. This variability may reflect differences in study populations, interventions, or other factors, emphasizing the need for cautious interpretation of pooled results and tailoring of clinical decision‐making to specific contexts. Issues affecting guideline adherence could also play a role in the effect sizes reported in the studies. For example, changes in guideline‐recommended care over the years, variations in techniques in surgical interventions, variations in hospital protocols or procedures, and referral bias in studies done in tertiary centers could all impact the effect sizes reported. Hence the absolute effect size reported in this study should be interpreted with caution.

### 4.2 Research and Practice Implications

This is the first systematic review and meta‐analysis of studies evaluating the impact of guideline adherence on survival in all cancer patients regardless of their disease site. This provides a knowledge database that researchers can build on in further analyzing the impact on guidelines for each disease site or to identify areas where research is lacking. The volume of research identified in this study by disease site corresponds with the global disease burden with more patients being diagnosed with breast, gastrointestinal, and gynecological cancers as opposed to head and neck or thoracic cancers. Furthermore, this systematic review serves as a resource for registry database developers or institutional record keepers to use to identify important variables that need to be captured in their databases (e.g., QOL data). The meta‐analysis also shows, with a low certainty of evidence, that adherence to CPG recommendations is associated with better survival in all the disease sites with the exception of gynecological cancers where the pooled analysis did not show an OS benefit in CPG adherence. Many of the studies that could not be pooled also showed similar trends in data. Given these findings, we recommend that guideline adherence should be encouraged in clinical practice to improve patient outcomes. There is also a need for more research to be done in disease sites where this is lacking. Studies should also be of a prospective study design with predefined variables and study endpoints rather than relying on data collected retrospectively. Given the cost and time associated in developing these guidelines, findings from this study provides stakeholders and policy makers with evidence that can be used in program planning and resource allocation.

## 5 Conclusion

This systematic literature review and meta‐analysis of studies in patients with cancer shows that adherence to CPG recommendations may improve OS, RFS and DFS. However, there remains some unexplained sources of high statistical heterogeneity. This study highlights the need for more work to be done in improving guideline implementation strategies and adherence to guideline recommendations should be encouraged in clinicians. Hence, before guideline developers start to invest more time and money into the new world of artificial intelligence enabled guidelines, it is imperative for us to evaluate the impact of the guidelines we currently have in use.

## Author Contributions

**Nofisat Ismaila:** conceptualization, data curation, formal analysis, investigation, methodology, project administration, resources, software, visualization, writing – original draft, writing – review and editing. **Lawrence Mbuagbaw:** supervision validation, writing – review and editing. **Jinhui Ma:** supervision validation, writing – review and editing. **Lehana Thabane:** supervision validation, writing – review and editing.

## Ethics Statement

The authors have nothing to report.

## Conflicts of Interest

The authors declare no conflicts of interest.

## Supporting information

Suplementary material_S3_CLEAN.

## Data Availability

The data collection forms and data extracted for this study are stored in the DistillerSR online platform. All other data that supports the findings from this study are available in the data supplement.
