# Supplementary material for: Clinical Practice Guidelines Adherence and the Impact on Health Outcomes in Oncology: A Systematic Review and Meta‐Analysis
Source: Clin Public Health Guidel. 2025 Jul 21;2(3):e70030. doi: 10.1002/gin2.70030 (PMC13563329; doi:10.1002/gin2.70030)

Clinical Practice Guidelines Adherence and the Impact on Health Outcomes in Oncology: A Systematic Review and Meta-analysis

APPENDIX A

Literature search strategy

OVID Medline Epub Ahead of Print, In-Process & Other Non-Indexed Citations, Ovid MEDLINE(R) Daily and Ovid MEDLINE(R) 1946 to December 18 2024

1	Practice Guideline.mp. or Practice Guideline/	37910
2	guideline/	11927
3	guidelines as Topic/	39537
4	practice guideline*.ti,ab.	35754
5	clinical guid*.ti,ab.	22771
6	1 or 2 or 3 or 4 or 5	126093
7	exp Neoplasms/	2869817
8	cancer*.ti,ab.	2193207
9	neoplasm*.ti,ab.	128557
10	malignan*.ti,ab.	598506
11	tumo?r*.ti,ab.	1813877
12	7 or 8 or 9 or 10 or 11	4077860
13	Guideline Adherence/	36242
14	"diffusion of innovation"/	16649
15	Practice Patterns, Physicians'/	65531
16	(guideline* adj3 (implement* or adher* or translat* or follow*)).ti,ab.	47576
17	practice pattern*.ti,ab.	10961
18	or/13-17	159011
19	6 and 12 and 18	1732

Embase <1974 to 2024 December 18>

1	practice guideline/	598513
2	guideline*.ti,ab.	839561
3	practice guid*.ti,ab.	53009
4	clinical guid*.ti,ab.	35038
5	or/1-4	1098945
6	exp neoplasm/	6187924
7	cancer*.ti,ab.	3456494
8	neoplasm*.ti,ab.	232024
9	malignan*.ti,ab.	1058302
10	tumo?r*.ti,ab.	2959898
11	or/6-10	7272782

12 guideline adherence.mp. 5175
13 (guideline* adj3 (implement* or adher* or translat* or follow*)).ti,ab. 74501
14 practice pattern*.ti,ab. 18690
15 or/12-14 93346
16 5 and 11 and 1516651
17 (guideline* adj3 (implement* or adher* or translat*)).ti,ab. 39430
18 12 or 14 or 17 58465
19 5 and 11 and 188819
20 limit 19 to english language 8648
21 limit 20 to human 8313
22 (editorial or note or letter erratum or short survey or conference).pt. or abstract report/ or letter/ or case study/ or protocol/ 9780888
23 21 not 22 4265

CINAHL DATABASE – October 2023

#	Query	Results
S12	S8 AND S10 AND S11	1,838
S11	TI (neoplasms or oncology or cancer or tumor or malignancy) OR AB (neoplasms or oncology or cancer or tumor or malignancy)	693,512
S10	S4 OR S5 OR S9	36,132
S9	TX guideline implementation	1,069
S8	S3 OR S6 OR S7	433,798
S7	AB guidelines or protocols or practice guideline or clinical practice guideline or recommendation	361,559
S6	TI guidelines or protocols or practice guideline or clinical practice guideline or recommendation	116,797
S5	TX practice patterns	17,561
S4	TX guideline adherence	18,934
S3	S1 OR S2	17,995
S2	AB practice guidelines or evidence-based practice guidelines or clinical practice guidelines	12,772
S1	TI practice guidelines or evidence-based practice guidelines or clinical practice guidelines	7,645

APA PsycInfo <1806 to December 2024 Week 2>

1	treatment guidelines/	10355
2	evidence based practice/	22397
3	guideline*.ti,ab.	83425
4	practice guid*.ti,ab.	5956
5	clinical guid*.ti,ab.	3846
6	or/1-5	106790
7	exp Neoplasms/	67329
8	cancer*.ti,ab.	77078
9	neoplasm*.ti,ab.	1441
10	malignan*.ti,ab.	7918
11	tumo?r*.ti,ab.	20116
12	or/7-11	101843
13	guideline adherence.mp.	2580
14	practice pattern*.ti,ab.	928
15	(guideline* adj3 (implement* or adher* or translat* or follow*)).ti,ab.	7749
16	or/13-15	10497
17	6 and 12 and 16	644

Cochrane Controlled Trials Register – December 2024

ID	Search	Hits
#1	Practice Guideline	18420
#2	clinical guideline	21675
#3	Guidelines	56810
#4	neoplasms OR cancer OR Oncology	268249
#5	tumor OR tumour OR carcinoma	122040
#6	precancerous OR malignant OR adenocarcinoma	38671
#7	Guideline Adherence OR Adherence	52683
#8	#1 OR #2 OR #3	66115
#9	#4 OR #5 OR #6	301257
#10	#8 AND #9	12559
#11	#10 and #7	1629

APPENDIX B: *Table 1 Characteristics of included studies (N=75)*

**Author last name and year**<br><br>**Study country**	**Study design**<br><br>**Institution(N)/Registry database**<br><br>**Patient population**	**Guideline used**<br><br>**Total N**	**Outcomes**<br><br>**Follow-up duration (mean or total*)**	**Risk of bias**<br><br>**NOS score**
**GASTROINTESTINAL**				
Han 2023<br><br>Europe	Retrospective cohort<br><br>Registry<br><br>Stage B hepatocellular carcinoma	APASL, EASL, and the AASLD<br><br>N=650	Adherence, survival<br><br>5 years	Low<br><br>8
Chen 2023<br><br>Asia	Retrospective cohort<br><br>Institution (1)<br><br>T4 Rectal Cancer	Chinese protocol for the diagnosis and treatment of CRC (2020 edition)<br><br>N=397	Adherence, survival<br><br>29 months	Low<br><br>8
Kumar 2023<br><br>North America	Retrospective cohort<br><br>Registry<br><br>Anal cancer	NCCN<br><br>N=4740	Adherence, survival<br><br>5 years	Low<br><br>8
Reni 2023<br><br>Europe	Prospective cohort<br><br>Institutions (45)<br><br>Pancreatic cancer	Italian Association of Medical Oncology (AIOM)<br><br>N=833	Adherence, survival<br><br>34.5 months	Low<br><br>8
Munir 2023<br><br>North America	Retrospective cohort<br><br>Registry<br><br>cholangiocarcinoma	NCCN<br><br>N=4519	Adherence, survival<br><br>5 years	Low<br><br>8
Kaslow 2022<br><br>North America	Retrospective cohort<br><br>Registry<br><br>Gastric cancers	NCCN<br><br>N=37659	Adherence, survival<br><br>12 years	Low<br><br>8
van den Berg 2021<br><br>Europe	Retrospective cohort<br><br>Institution (7)<br><br>Stage III colon cancer	Not specified<br><br>N=575	Adherence, survival<br><br>47 months	Moderate<br><br>7
Carrasco-Pena, 2020<br><br>Europe	Retrospective cohort<br><br>Granada and Girona cancer registries<br><br>Colorectal cancer	Integrated Healthcare Process for Colorectal Cancer (Granada and Onco-Guide of Colon and Rectum (Girona)	Adherence, survival<br><br>5 years	Low<br><br>8

Author last name and year<br><br>Study country	Study design<br><br>Institution(N)/Registry database<br><br>Patient population	Guideline used<br><br>Total N	Outcomes<br><br>Follow-up duration (mean or total*)	Risk of bias<br><br>NOS score
		N=1050		
Chow, 2020<br><br>North America	Retrospective cohort<br><br>Kentucky Cancer Registry<br><br>locally advanced stage II/III colon cancer	NCCN<br><br>N=1404	Adherence, survival<br><br>5 years	Moderate<br><br>7
Eid, 2019<br><br>Europe	Pre-Post<br><br>Institution (1)<br><br>Subperitoneal rectal cancer	French national health authority<br><br>N=604	Adherence, survival<br><br>75.9 months (before guidelines)<br>43.1 months (after guidelines)	Moderate<br><br>6
Jaap, 2018<br><br>North America	Retrospective cohort<br><br>National Cancer Database<br><br>Stage I/II pancreatic cancer	NCCN<br><br>N= 52450	Adherence, survival<br><br>8 years*	Moderate<br><br>6
van Vuuren, 2018<br><br>Europe	Retrospective cohort<br><br>Institution (2)<br><br>Rectal adenocarcinoma	National Dutch guidelines<br><br>N=108	Adherence, survival<br><br>18 months	Moderate<br><br>6
Bagante, 2018<br><br>North America	Retrospective cohort<br><br>National Cancer Database<br><br>Biliary tract cancers	NCCN<br>Comprehensive Cancer Center Middle Netherlands (CCCMN)<br>N=176536	Adherence, survival<br><br>10 years*	Moderate<br><br>6
Zhao, 2018<br><br>North America	Retrospective cohort<br><br>Texas Cancer Registry/Medicare<br><br>stage II and III colon cancer patients ≥66 years	NCCN<br><br>N=6028	Adherence, survival<br><br>5 years*	Moderate<br><br>7
Adelson, 2017<br><br>Australia	Retrospective cohort<br><br>South Australia Clinical Cancer Registry<br><br>Stage C colon and stage B and C rectal cancer	Australian Cancer Council guidelines<br><br>N=4237	Adherence, survival<br><br>5 years	Low<br><br>8
Guerrero, 2017<br><br>North America	Retrospective cohort<br><br>Institution (1)	American College of Surgeons<br><br>N=438	Adherence, survival<br><br>38 months for guideline compliant; 45.8 months	Moderate<br><br>7

Author last name and year<br><br>Study country	Study design<br><br>Institution(N)/Registry database<br><br>Patient population	Guideline used<br><br>Total N	Outcomes<br><br>Follow-up duration (mean or total*)	Risk of bias<br><br>NOS score
	Stage III colon cancer		for non-guideline compliant	
Guarino, 2017<br><br>Europe	Retrospective cohort<br><br>Progetto Epatocarcinoma Campania<br><br>Hepatocellular carcinoma	Barcelona Clinic Liver Cancer guidelines<br><br>N=1008	Adherence, survival<br><br>25 months	Moderate<br><br>5
Kikuchi, 2017<br><br>Europe	Retrospective cohort<br><br>Institution (1)<br><br>Hepatocellular carcinoma	Barcelona Clinic Liver Cancer guidelines<br><br>N=364	Adherence, survival<br><br>19 months	Moderate<br><br>7
Guarino, 2015<br><br>Europe	Prospective cohort<br><br>Institution (1)<br><br>Hepatocellular carcinoma	Barcelona Clinic Liver Cancer guidelines<br><br>N=92	Adherence, survival<br><br>3 years	Moderate<br><br>6
Worhunsky, 2015<br><br>North America	Retrospective cohort<br><br>California Cancer Registry<br><br>Gastric cancer	NCCN<br><br>N=10093	Adherence, survival<br><br>5 years	Moderate<br><br>7
Boland, 2013<br><br>North America	Retrospective cohort<br><br>National Cancer Database<br><br>Stage III and high-risk stage II colon cancer	NCCN<br><br>N=167434	Adherence, survival<br><br>NR	Moderate<br><br>6
Abrams, 2012<br><br>North America	Phase 3 RCT<br><br>Institution<br><br>Adenocarcinoma of the pancreas	Not Specified<br><br>N=416	Adherence, survival<br><br>1.5 years and 4.7 years on surviving patients	Low
Van Gils, 2011<br><br>Europe	Retrospective cohort<br><br>Institution (19)<br><br>Stage III colon cancer	National Dutch Guidelines<br><br>N=423	Adherence, survival<br><br>NR	Moderate<br><br>7
Manchon-Walsh, 2011<br><br>Europe	Retrospective cohort<br><br>Institution<br><br>Rectal cancer	Catalonia Colorectal cancer guidelines<br><br>N=1229	Adherence, survival<br><br>2 years	Moderate<br><br>7

Author last name and year<br><br>Study country	Study design<br><br>Institution(N)/Registry database<br><br>Patient population	Guideline used<br><br>Total N	Outcomes<br><br>Follow-up duration (mean or total*)	Risk of bias<br><br>NOS score
**BREAST**				
Song, 2022<br><br>Asia	Retrospective cohort<br><br>Registry (2)<br>Universiti Malaya Breast Cancer Registry, Ramsay Sime Darby Breast Cancer Registry<br><br>Women with stage I-III breast cancer	Malaysian Clinical Practice Guidelines for Management of Breast Cancer Second Edition<br><br>N=3100	Adherence, survival<br><br>5 years	Moderate<br><br>7
Wimmer 2019<br><br>Europe	Retrospective cohort<br><br>Tumor Centre<br>Regensburg Registry<br><br>Patients with invasive breast cancer after breast-conserving therapy (BCT)	German national consensus S3 guideline<br><br>N=6370	Adherence, survival<br><br>6.3 years	Low<br><br>8
Kim, 2019<br><br>Asia	Retrospective cohort<br><br>Institution (1)<br><br>patients with premenopausal HR-positive/HER2-negative MBC	Not specified<br><br>N =272	Adherence, survival<br><br>38.7 months	Moderate<br><br>7
Kreienberg 2018<br><br>Europe	Controlled before-after study<br><br>Institution (17)<br><br>Patients with primary breast cancer	German national consensus S3 guideline<br><br>N=8323	Adherence, survival<br><br>5 years*	Moderate<br><br>7
Wollschlager, 2018<br><br>Europe	Retrospective cohort<br><br>Institution (17)<br><br>BRENDA DATABASE<br><br>Comorbid breast cancer patients	German national consensus S3 guideline<br><br>N=2137	Adherence, survival<br><br>5.7 years	Low<br><br>9
Rocque, 2018<br><br>North America	Retrospective cohort<br><br>SEER-Medicare database<br><br>Women with de novo stage IV metastatic breast cancer	NCCN<br><br>N=988	Adherence, survival<br>Costs to Medicare/ healthcare utilization<br><br>2.1 years	Moderate<br><br>7
Andreano, 2017<br><br>Europe	Retrospective cohort<br><br>Lomdardy, Italy Cancer Registry database<br><br>Women with an epithelial breast cancer	National Collaborating Centre for Cancer, Cardiff and ESMO<br><br>N=6333	Adherence, survival<br><br>5.6 years	Low<br><br>8

Author last name and year<br><br>Study country	Study design<br><br>Institution(N)/Registry database<br><br>Patient population	Guideline used<br><br>Total N	Outcomes<br><br>Follow-up duration (mean or total*)	Risk of bias<br><br>NOS score
Jacke, 2015<br><br>Europe	Retrospective cohort<br><br>Institution (3)<br><br>Women with primary breast cancer	German national consensus guideline (S1 & S3)<br><br>N= 877	Adherence, survival<br><br>5 years	Moderate<br><br>6
Wolters, 2015<br><br>Europe	Retrospective cohort<br><br>Institution (17)<br><br>Women with breast cancer	Not specified<br><br>N=9061	Adherence, survival<br><br>10 years*	Moderate<br><br>6
Schwenter, 2013<br><br>Europe	Retrospective cohort<br><br>Institution (17)<br><br>BRENDA DATABASE<br><br>Women with TNBC	German national consensus guideline (S3)<br><br>N=844	Adherence, survival<br><br>15 years*	Moderate<br><br>7
Sacerdote 2013<br><br>Europe	Retrospective cohort<br><br>Institution (1)<br><br>Patients with incident breast cancer	Piedmont guidelines<br><br>N=1092	Adherence, quality of care, mortality<br><br>2 years*	Moderate<br><br>7
Schwentner, 2013<br><br>Europe	Retrospective cohort<br><br>Institution (17)<br><br>BRENDA DATABASE<br><br>histologically confirmed invasive breast cancer	German national consensus S3 guideline<br><br>N=9433	Adherence, survival<br><br>15 years*	Moderate<br><br>6
Schwentner, 2012<br><br>Europe	Retrospective cohort<br><br>Institution (17)<br><br>BRENDA DATABASE<br><br>Histologically confirmed invasive breast cancer	German national consensus S3 guideline<br><br>N= 5292	Adherence, survival<br><br>10 years*	Moderate<br><br>7
Schwentner, 2012<br><br>Europe	Retrospective cohort<br><br>Institution (17)<br><br>BRENDA DATABASE<br><br>Histologically confirmed invasive breast cancer with TNBC	German national consensus S3 guideline<br><br>N= 3658	Adherence, survival<br><br>5 years	Low<br><br>8
Wockel, 2010<br><br>Europe	Retrospective cohort<br><br>Institution (17)	German national consensus S3 guideline	Adherence, survival<br><br>5 years	Moderate<br><br>6

Author last name and year<br><br>Study country	Study design<br><br>Institution(N)/Registry database<br><br>Patient population	Guideline used<br><br>Total N	Outcomes<br><br>Follow-up duration (mean or total*)	Risk of bias<br><br>NOS score
	BRENDA DATABASE<br><br>Histologically confirmed invasive breast cancer	N=3976		
Varga, 2010<br><br>Europe	Retrospective cohort<br><br>Institution (17)<br><br>BRENDA DATABASE<br><br>Breast cancer patients (the very young under 35 years and the younger patients between 36 and 55, all premenopausal)	German national consensus S3 guideline<br><br>N= 1778	Adherence, survival<br><br>mean follow-up was 59.6 months for patients diagnosed at <35 years and 62.1 months for patients diagnosed at 35–55 years.	Low<br><br>8
Wockel, 2010<br><br>Europe	Retrospective cohort<br><br>Institution (17)<br><br>BRENDA DATABASE<br><br>histologically confirmed invasive breast cancer	German national consensus S3 guideline<br><br>N=2231	Adherence, survival<br><br>13 years*	Moderate<br><br>6
**GYNECOLOGICAL**				
Kamat 2023<br><br>USA	Retrospective cohort<br><br>SEER database<br><br>Older Women With Advanced Epithelial Ovarian Cancer	NCCN<br><br>N=3237	Adherence, survival<br><br>22 months	Low<br><br>8
Jochum, 2021<br><br>Europe	Retrospective cohort<br><br>Institution (12)<br><br>Invasive epithelial ovarian cancer	ESMO-European Society of Gynecologic Oncology (ESGO) ovarian cancer guideline<br><br>N= 1463	Adherence, survival<br><br>30 months	Low<br><br>8
Mandato, 2021<br><br>Europe	Pre—post<br><br>Institution (1)<br><br>Endometrial	Provincial guidelines (Reggio Emilia)<br><br>N=409	Adherence, survival<br><br>NR	Low<br><br>8
Pfaendler, 2019<br><br>North America	Retrospective cohort<br><br>California Cancer Registry<br><br>Stage IB-IIA Cervical Cancer	NCCN<br><br>N=6063	Adherence, survival<br><br>8.7 years	Low<br><br>8

Author last name and year<br><br>Study country	Study design<br><br>Institution(N)/Registry database<br><br>Patient population	Guideline used<br><br>Total N	Outcomes<br><br>Follow-up duration (mean or total*)	Risk of bias<br><br>NOS score
Levinson 2019<br><br>North America	Retrospective cohort<br><br>Chart review<br><br>HIV infected women with gynecologic cancers	NCCN<br><br>N=57	Adherence, survival<br><br>48 months	Moderate<br><br>6
Shigeta, 2017<br><br>Asia	Pre-Post<br><br>Japan Society of Obstetrics and Gynecology (JSOG) cancer registration system<br><br>Endometrial cancer	JSGO<br><br>N= 65,241	Adherence, survival<br><br>12 years*	Moderate<br><br>6
Chiew, 2017<br><br>Australia	Retrospective cohort<br><br>Southwest Sydney Local Health District and Sydney Local Health District Clinical Cancer Registry<br><br>Cervical cancer	NCCN, JSGO, ESMO, NICE<br><br>N=208	Adherence, survival<br><br>5 years	Moderate<br><br>6
Lee, 2015<br><br>Asia	Retrospective cohort<br><br>Seoul National University Hospital registry database<br><br>Ovarian cancer	NCCN<br><br>N=266	Adherence, survival<br><br>8.4 years	Moderate<br><br>7
Sijmons, 2007<br><br>Europe	Retrospective cohort<br><br>Regional Cancer Registry (central Netherlands)<br><br>Early-stage epithelial ovarian carcinoma	Comprehensive Cancer Center Middle Netherlands<br><br>N=125	Adherence, survival<br><br>734.6 person years	Low<br><br>8
Lankveld, 2006<br><br>Europe	Retrospective cohort<br><br>Regional Cancer Registry Netherlands<br><br>Early-stage endometrial cancer	Comprehensive Cancer Center Middle Netherlands<br><br>N=359	Adherence, survival<br><br>68.8 months	Low<br><br>8
Howell, 2000<br><br>North America	Retrospective cohort<br><br>SEER database<br><br>Cervical cancer	International Federation of Gynecology and Obstetrics<br><br>N=5952	Adherence, survival<br><br>7 years*	Moderate<br><br>6
**GENITOURINARY**				
Sato 2023	Retrospective cohort	EAU	Adherence, survival	Low

Author last name and year<br><br>Study country	Study design<br><br>Institution(N)/Registry database<br><br>Patient population	Guideline used<br><br>Total N	Outcomes<br><br>Follow-up duration (mean or total*)	Risk of bias<br><br>NOS score
Asia	Institution (10)<br><br>Bladder cancer	N=1204	56 months	8
Ulvskog 2023<br><br>Europe	Before and after<br><br>Registry<br><br>Penile cancer	Swedish guideline<br><br>N=274	Adherence ,survival<br><br>5 years	Low<br><br>8
Khayyat, 2021<br><br>Europe	Retrospective cohort<br><br>Institution (2)<br><br>Bladder cancer	European Association of Urology<br><br>N=88	Adherence, RR, PR, and mortality rate.<br><br>3 years	Moderate<br><br>7
Serrano 2021<br><br>Europe	Prospective cohort<br><br>Geriatric oncology clinics (19)<br><br>Prostate cancer	French Association of Urology Guideline International Society of Geriatric Oncology and European Association of Urology<br><br>N= 275	Adherence, survival<br><br>44 months	Moderate<br><br>7
Cindolo, 2019<br><br>Europe	Retrospective cohort<br><br>Institution<br><br>Penile cancer	European Association of Urology<br><br>N=425	Adherence, survival<br><br>17 months	Moderate<br><br>7
Sorenby, 2019<br><br>Europe	Pre-post<br><br>Institution (1)<br><br>Non-muscle invasive bladder cancer	Standardized treatment protocol<br><br>N=275	Adherence, survival<br><br>36 months	Moderate<br><br>7
Paffenholz, 2017<br><br>Europe	Retrospective cohort<br><br>Institution (1)<br><br>Testicular cancer	European Association of Urology<br><br>N=131	Adherence, survival<br><br>9 months	Moderate<br><br>7
Lammers, 2014<br><br>Europe	Retrospective cohort<br><br>Institution (41)<br><br>Bladder cancer	European Association of Urology, American Urological Association, European Organization for Research and Treatment of Cancer, Club Urologico Espanol de Tratamiento Oncologico	Adherence, survival<br><br>29.6 months	Moderate<br><br>5

Author last name and year<br><br>Study country	Study design<br><br>Institution(N)/Registry database<br><br>Patient population	Guideline used<br><br>Total N	Outcomes<br><br>Follow-up duration (mean or total*)	Risk of bias<br><br>NOS score
		N=1045		
**HEAD & NECK CANCERS**				
Chen 2024<br><br>USA	Retrospective cohort<br><br>National Cancer Data Base<br><br>Locally advanced laryngeal and hypopharyngeal cancer	NCCN<br><br>N=2823	Adherence, survival<br><br>5 years	Low<br><br>8
Frisco 2023<br><br>North America	Retrospective cohort<br><br>SEER Data Base<br><br>Thyroid cancer	ATA<br><br>N=2898	Adherence, survival<br><br>5 years	Low<br><br>8
Cohen 2022<br><br>North America	Retrospective cohort<br><br>National Cancer Data Base<br><br>stage III and IVA HNC	NCCN<br><br>N=100,074	Adherence, survival<br><br>5 years	Low<br><br>8
McMullin 2022<br><br>North America	Retrospective cohort<br><br>National Cancer Data Base<br><br>Thyroid cancer	ATA<br><br>N=3421	Adherence, survival<br><br>5 years	Low<br><br>8
Roden, 2019<br><br>North America	Retrospective cohort<br><br>Institution (1)<br><br>HNC	NCCN<br><br>N=159	Adherence, survival<br><br>391 days	Moderate<br><br>7
Schwam, 2016<br><br>North America	Retrospective cohort<br><br>National Cancer Data Base<br><br>Nasopharyngeal Carcinoma	NCCN<br><br>N=1741	Adherence, survival<br><br>46.8 months	Moderate<br><br>7
Adam, 2015<br><br>North America	Retrospective cohort<br><br>National Cancer Data Base<br><br>Thyroid cancer	NCCN<br>American Thyroid Association<br><br>N=39687	Adherence, survival<br><br>75 months	Moderate<br><br>7
Verbeek, 2015<br><br>Europe	Retrospective cohort<br><br>Institution (2)	ATA<br><br>N=184	Adherence, survival<br><br>NR	Moderate<br><br>6

Author last name and year<br><br>Study country	Study design<br><br>Institution(N)/Registry database<br><br>Patient population	Guideline used<br><br>Total N	Outcomes<br><br>Follow-up duration (mean or total*)	Risk of bias<br><br>NOS score
	Thyroid cancer			
Aarts, 2005<br><br>Europe	Retrospective cohort<br><br>Institution (1)<br><br>Tongue and floor of mouth squamous carcinoma	Comprehensive Cancer Center Middle Netherlands<br><br>N=217	Adherence, survival<br><br>5 years	Low<br><br>8
**THORACIC CANCERS**				
Tissera 2024<br><br>Australia	Retrospective cohort<br><br>Registry database<br><br>Stage I-IV NSCLC	NR<br><br>N=11073	Adherence, survival<br><br>5 years	Low<br><br>8
Schneider 2022<br><br>North America	Retrospective cohort<br><br>Registry database<br><br>Node-negative NSCLC	NCCN<br><br>N=2020	Adherence, survival<br><br>18.7 months	Low<br><br>8
John, 2021<br><br>North America	Retrospective cohort<br><br>Registry Database<br><br>Advanced NSCLC	NCCN<br><br>N=28784	Adherence, survival<br><br>11.7 months	Moderate<br><br>7
Duggan, 2016<br><br>Australia	Retrospective cohort<br><br>SW Sydney Clinical Cancer Registry<br><br>Stage I-IIIb NSCLC	Australian Cancer Council guidelines<br><br>N= 592	Adherence, survival<br><br>54 months	Moderate<br><br>7
Yue, 2014<br><br>Asia	Retrospective cohort<br><br>Institution (1)<br><br>NSCLC	NCCN<br>American College of Surgeons Oncology Group, OSI Pharmaceutical RADIANT trial and IASLC<br><br>N=2711	Adherence, survival<br><br>96 months*	Low<br><br>8
**MELANOMA**				
Erickson Foster, 2008<br><br>North America	Retrospective cohort<br><br>Rush University Institutional Cancer Registry database<br><br>Melanoma	NCCN<br><br>N=297	Adherence, survival<br><br>51 months	Moderate<br><br>7

Abbreviations: NCCN, National Comprehensive Cancer Network; APASL, Asian Pacific Association for the Study of the Liver; EASL, European Association for the Study of the Liver; AASLD, American Association for the Study of Liver Diseases; RCT, Randomized control trial; RFS, Recurrence free survival; PFS, Progression free survival; OS, Overall survival; ESMO, European Society of Medical Oncology; ATA, American Thyroid Association; BRENDA, breast cancer care under evidence-based guidelines; MBC, Metastatic breast cancer; NSCLC, Non-Small Cell Lung Cancer; HR, Hormone receptor; NICE, National Institute for Health and Care Excellence; SEER, Surveillance, Epidemiology, and End Results; CCMN, Comprehensive Cancer Center Middle Netherlands; NMIBC, non-muscle-invasive bladder cancer; TURB, transurethral resection of the bladder; N, number.

APPENDIX C: List of excluded studies grouped by reasons for exclusion

Does not include a study population of interest (n=10)

1. Keikes, Lotte, van Oijen, Martijn G. H., Lemmens, Valery E. P. P., Koopman, Miriam, Punt, Cornelis J. A.. Evaluation of Guideline Adherence in Colorectal Cancer Treatment in The Netherlands: A Survey Among Medical Oncologists by the Dutch Colorectal Cancer Group. Clinical Colorectal Cancer. 2018. 17:58-64
2. Kagramanov, Dalia, Sutradhar, Rinku, Lau, Cindy, Yao, Zhan, Pole, Jason D., Baxter, Nancy N., Gupta, Sumit, Nathan, Paul C.. Impact of the model of long-term follow-up care on adherence to guideline-recommended surveillance among survivors of adolescent and young adult cancers. Cancer Medicine. 2021. 10:5078-5087
3. Kockerling, F., Schug-Pass, C., Bittner, R., Kuthe, A., Stechemesser, B., Lorenz, R., Koch, A., Reinpold, W., Niebuhr, H., Hukauf, M.. Laparo-endoscopic versus open recurrent inguinal hernia repair: should we follow the guidelines?. Surgical Endoscopy. 2017. 31:3168-3185
4. Abrahao, Renata, Keegan, Theresa H M, Maguire, Frances B, Li, Qian, Malogolowkin, Marcio H, Wong, Samantha, Thorpe, Steven W, Carr-Asher, Janai R, Midboe, Amanda M, Randall, R Lor, Alvarez, Elysia M. Receipt of Guideline-Concordant Care Is Associated With Improved Survival in Patients With Osteosarcoma in California: A Population-Based Analysis.. JCO oncology practice. 2024//. 20:1064
5. Tomasone, Jennifer R., Kauffeldt, Kaitlyn D., Chaudhary, Rushil, Brouwers, Melissa C.. Effectiveness of guideline dissemination and implementation strategies on health care professionals' behaviour and patient outcomes in the cancer care context: a systematic review. Implementation Science. 2020. 15:1-18
6. Findlay, Merran, Bauer, Judith D., Dhaliwal, Rupinder, de van der Schueren, Marian, Laviano, Alessandro, Widaman, Adrianne, Martin, Lisa, Day, Andrew G., Gramlich, Leah M.. Translating Evidence-Based Guidelines into Practice—Are We Getting It Right? A Multi-Centre Prospective International Audit of Nutrition Care in Patients with Foregut Tumors (INFORM). Nutrients. 2020. 12:3808
7. Bierbaum, Mia, Rapport, Frances, Arnolda, Gaston, Nic Giolla Easpaig, Brona, Lamprell, Klay, Hutchinson, Karen, Delaney, Geoff P., Liauw, Winston, Kefford, Richard, Olver, Ian, Braithwaite, Jeffrey. Clinicians' attitudes and perceived barriers and facilitators to cancer

treatment clinical practice guideline adherence: a systematic review of qualitative and quantitative literature. Implementation Science. 2020. 15:1-24
8. Trevisiol, Chiara, Cinquini, Michela, Fabricio, Aline S. C., Gion, Massimo, Rutjes, Anne W. S.. Insufficient uptake of systematic search methods in oncological clinical practice guideline: a systematic review. BMC Medical Research Methodology. 2019. 19:N.PAG-N.PAG
9. Peabody, John W, Cruz, Jamielyn D C, Ganesan, Divya, Paculdo, David, Critchley-Thorne, Rebecca J, Wani, Sachin, Shaheen, Nicholas J 2024//. A Randomized Controlled Study on Clinical Adherence to Evidence-Based Guidelines in the Management of Simulated Patients With Barrett's Esophagus and the Clinical Utility of a Tissue Systems Pathology Test: Results From Q-TAB. Clinical and translational gastroenterology, 15(1): e00644.
10. Asklid, A, Nilsson, M P, Engellau, J, Kristensen, I, Blomstrand, M, Frojd, C, Agrup, M, Flejmer, A, Martinsson, U, Svard, A-M, Almhagen, E, Embring, A 2024//. Reirradiation in Paediatric Tumours of the Central Nervous System: Outcome and Side Effects After Implementing National Guidelines. Clinical oncology (Royal College of Radiologists (Great Britain)), 37(#issue#): 103667.

Does not include intervention of interest (n=21)

11. Ming, Jie, Zhu, Jing-Qiang, Zhang, Hao, Sun, Hui, Wang, Jun, Cheng, Ruo-Chuan, Xie, Lei, Li, Xing-Rui, Tian, Wen, Huang, Tao. A multicenter, prospective study to observe the initial management of patients with differentiated thyroid cancer in China (DTCC study). BMC Endocrine Disorders. 2021. 21:1-12
12. Twomey, Rosie, Matthews, T. Wayne, Nakoneshny, Steven C., Schrag, Christiaan, Chandarana, Shamir P., Matthews, Jennifer, McKenzie, David, Hart, Robert D., Li, Na, Dort, Joseph C., Sauro, Khara M.. From Pathways to Practice: Impact of Implementing Mobilization Recommendations in Head and Neck Cancer Surgery with Free Flap Reconstruction. Cancers. 2021. 13:2890
13. Zemanova, Milada, Jakopovic, Marko, Stanic, Karmen, Łazar-Poniatowska, Małgorzata, Vrankar, Martina, Rusu, Petronela, Ciuleanu, Tudor, Radosavljevic, Davorin, Bogos, Krisztina, Nawrocki, Sergiusz. Treatment patterns and real-world evidence for stage III non-small cell lung cancer in Central and Eastern Europe. Radiology & Oncology. 2020. 54:447-454
14. Thiels, Cornelius A., Hanson, Kristine T., Habermann, Elizabeth B., Boughey, Judy C., Grotz, Travis E.. Integrated cancer networks improve compliance with national guidelines and outcomes for resectable gastric cancer. Cancer (0008543X). 2020. 126:1283-1294
15. Perlow, Haley K., Ramey, Stephen J., Cassidy, Vincent, Kwon, Deukwoo, Farnia, Benjamin, Nicolli, Elizabeth, Samuels, Michael A., Freedman, Laura, Elsayyad, Nagy, Yechieli, Raphael, Samuels, Stuart E.. Disparities in adherence to head and neck cancer follow-up guidelines. Laryngoscope. 2019. 129:2303-2308
16. Jung, Kyung Yoon Kylie, Shadbolt, Bruce, Rezo, Angela. Temporal impact of the publication of guidelines and randomised evidence on the adoption of hypofractionated whole breast radiotherapy for early-stage breast cancer. Journal of Medical Imaging & Radiation Oncology. 2019. 63:530-537

17. Chang, Steven, Sanii, Ryan, Chaudhary, Hamad, Lewis, Carol, Seidman, Michael, Yaremchuk, Kathleen. Evaluation of early oral cavity cancer treatment quality at a single institution. Laryngoscope. 2019. 129:1816-1821
18. Chen, Pei-Yan, Fang, Ai-Ping, Wang, Xiao-Yan, Lan, Qiu-Ye, Liao, Gong-Cheng, Liu, Zhao-Yan, Zhang, Dao-Ming, Zhang, Yao-Yun, Chen, Yu-Ming, Zhu, Hui-Lian. Adherence to the Chinese or American Dietary Guidelines is Associated with a Lower Risk of Primary Liver Cancer in China: A Case-Control Study. Nutrients. 2018. 10:1113
19. Leveridge, Michael J., Siemens, D. Robert, Brennan, Kelly, Izard, Jason P., Karim, Safiya, An, Howard, Mackillop, William J., Booth, Christopher M.. Temporal trends in management and outcomes of testicular cancer: A population-based study. Cancer (0008543X). 2018. 124:2724-2732
20. Pricolo, Victor E., Bonvini, Matteo, Abelli, Carlo F.. Patterns of care for anal cancer in the United States - a comparison between academic and community cancer centers. BMC Cancer. 2018. 18:1-5
21. Chen, Jonathan, Wu, Xian, Christos, Paul J., Formenti, Silvia, Nagar, Himanshu. Practice patterns and outcomes for patients with node-negative hormone receptor-positive breast cancer and intermediate 21-gene Recurrence Scores. Breast Cancer Research. 2018. 20:N.PAG-N.PAG
22. Hull, Leland E., Lynch, Julie A., Berse, Brygida B., DuVall, Scott L., Chun, Danielle S., Venne, Vicki L., Efimova, Olga V., Icardi, Michael S., Kelley, Michael J.. Clinical Impact of 21-Gene Recurrence Score Test Within the Veterans Health Administration: Utilization and Receipt of Guideline-Concordant Care. Clinical Breast Cancer. 2018. 18:135-143
23. Midura, Emily, Jung, Andrew, Daly, Meghan, Hanseman, Dennis, Davis, Bradley, Shah, Shimul, Paquette, Ian, Midura, Emily F., Jung, Andrew D., Daly, Meghan C., Hanseman, Dennis J., Davis, Bradley R., Shah, Shimul A., Paquette, Ian M.. Cancer Center Volume and Type Impact Stage-Specific Utilization of Neoadjuvant Therapy in Rectal Cancer. Digestive Diseases & Sciences. 2017. 62:1906-1912
24. Besse, Kees, Vernooij-Dassen, Myrra, Vissers, Kris, Engels, Yvonne. The Impact of a National Guideline on the Management of Cancer Pain on the Practice of Pain Assessment and Registration. Pain Practice. 2016. 16:148-153
25. Gross-Erne M., Zimmerli L., Bassetti S., Li Q., Feller A., Mingrone W. 2023//. A long-term retrospective observational study at a medium-sized medical oncology service in Switzerland: comparison of overall survival with a national cohort and adherence to treatment guidelines Swiss Medical Weekly, 153(7): 40091
26. Hernando-Requejo, Ovidio, Olombrada, Maria Victoria Torres, Bravo, Irene Alda, Moreno, Leire Arbea, Lopez-Campos, Fernando, Gonzalez, Mercedes Lopez, Martin, Margarita Martin, Macias, Virginia Morillo, De la Pinta, Carolina 2024//. Current landscape of gastrointestinal radiation oncology in Spain: a multicenter real-life survey and comparison with key clinical guidelines. Reports of practical oncology and radiotherapy : journal of Greatpoland Cancer Center in Poznan and Polish Society of Radiation Oncology, 29(3): 340.
27. Jacob, Jake, Millien, Valentine, Berger, Scott, Hernaez, Ruben, Ketwaroo, Gyanprakash A, Flores, Avegail G, Hou, Jason K, Jarbrink-Sehgal, Maria E, Khalaf, Natalia I, Rosen, Daniel G, El-Serag, Hashem B, Tan, Mimi C 2024//. Improving Adherence to Clinical Practice

Guidelines for Managing Gastric Intestinal Metaplasia Among Gastroenterologists at a US Academic Institution. Journal of clinical gastroenterology, 58(5): 432.
28. Cappellacci F., Canu G.L., Noli E., Argiolas A., Peis G., Lai M.L., Calo P.G., Medas F. 2024//. Changes in Clinical Practice in Adherence to the 2014 American Thyroid Association Guidelines on Thyroid Cancer: A Retrospective Study from a Tertiary Referral Center Journal of Personalized Medicine, 14(7): 727.
29. Lee R., Ku M., Je N.K. 2024//. Adherence to antiemetic guidelines in solid cancer patients receiving highly emetogenic chemotherapy in Korea Supportive Care in Cancer, 32(3): 190.
30. Bischof, D. A., Dodson, R., Jimenez, M. C., Behman, R., Cocieru, A., Blazer, D. G., Fisher, S. B., Squires, M. H., Kooby, D. A., Maithel, S. K., et al. 2015. Adherence to Guidelines for Adjuvant Imatinib Therapy for GIST: a Multi-institutional Analysis Journal of gastrointestinal surgery, 19(6): 1022-1028.
31. Ebben, Kees C W J, de Kroon, Cornelis D, Schmeink, Channa E, van der Hel, Olga L, van Vegchel, Thijs, Moncada-Torres, Arturo, de Hingh, Ignace H J T, van der Werf, Jurrian 2023//. A novel method for continuous measurements of clinical practice guideline adherence. Learning health systems, 7(4): e10384.

Does not include outcomes of interest (n=9)

32. Boac, Bernadette M., Xiong, Yin, Apte, Sachin M., Wenham, Robert M., Shahzad, Mian M., Munroe, Donald G., Lancaster, Johnathan M., Chon, Hye Sook. Adherence to practice guidelines is associated with reduced referral times for patients with ovarian cancer. American Journal of Obstetrics & Gynecology. 2018. 218:436.e1-436.e7
33. Heins, Marianne J., de Jong, Judith D., Spronk, Inge, Ho, Vincent K. Y., Brink, Mirian, Korevaar, Joke C.. Adherence to cancer treatment guidelines: influence of general and cancer-specific guideline characteristics. European Journal of Public Health. 2017. 27:616-620
34. Kahn, Ben, Freeland, Zachary, Gopal, Purva, Agrawal, Deepak, Mayorga, Christian, Mithani, Rozina, Skinner, Celette, Halm, Ethan, Singal, Amit, Mayorga, Christian A., Skinner, Celette Sugg, Halm, Ethan A., Singal, Amit G.. Predictors of guideline concordance for surveillance colonoscopy recommendations in patients at a safety-net health system. Cancer Causes & Control. 2015. 26:1653-1660
35. Xu, Jian-Yi, Vena, Jennifer E., Whelan, Heather K., Robson, Paula J.. Impact of adherence to cancer-specific prevention recommendations on subsequent risk of cancer in participants in Alberta's Tomorrow Project. Public Health Nutrition. 2019. 21:235-245
36. Jones, Petra, Cade, Janet E., Evans, Charlotte E. L., Hancock, Neil, Greenwood, Darren C.. Does adherence to the World Cancer Research Fund/American Institute of Cancer Research cancer prevention guidelines reduce risk of colorectal cancer in the UK Women's Cohort Study?. British Journal of Nutrition. 2018. 119:340-348
37. Bernicker, Eric H., Xiao, Yan, Abraham, Anup, Yang, Baiyu, Croix, Denise A., Redpath, Stella, Engstrom-Melnyk, Julia, Shah, Roma, Madala, Jaya, Allen, Timothy C.. Adherence to National Comprehensive Cancer Network ALK Testing Guidelines for Patients with Advanced Non-Small Cell Lung Cancer in U.S. Community Medical Centers. Oncologist. 2021. 26:e1050-e1057

38. Guo, Xiaoyue M., Cowan, Matthew, Pyrzak, Adam, Shulman, Lee, Barber, Emma L.. Impact of a Structured Early Detection Program on Adherence to Guidelines for Risk-Reducing Surgery in BRCA1/2 Carriers. Journal of Gynecologic Surgery. 2020. 36:262-266
39. Kim, Hee Kyung, Lee, Soo-Hyeon, Kim, Yu Jin, Park, Song Ee, Lee, Han Sang, Lim, Sung Won, Cho, Jang Ho, Kim, Ji-Yeon, Ahn, Jin Seok, Im, Young-Hyuck, Yu, Jong Han, Park, Yeon Hee. Does guideline non-adherence result in worse clinical outcomes for hormone receptor-positive and HER2-negative metastatic breast cancer in premenopausal women?: result of an institution database from South Korea. BMC Cancer. 2019. 19:1-8
40. Lainez, Nuria, Lecumberri, Maria J., Garcia-Donas, Jesus, Esteban, Emilio, Puente, Javier, Saez, M. Isabel, Gallardo, Enrique, Pinto-Marin, Alvaro, Vazquez-Estevez, Sergio, Leon, Luis, Garcia-Carbonero, Iciar, Suarez-Rodriguez, Cristina, Molins, Carmen, Climent-Duran, Miguel A., Lazaro-Quintela, Martin, Gonzalez del Alba, Aranzazu, Mendez-Vidal, Maria Jose, Chirivella, Isabel, Afonso, Francisco J., Lopez-Brea, Marta, Sala-Gonzalez, Nuria, Domenech, Montserrat, Basterretxea, Laura, Santander-Lobera, Carmen, Gil-Arnaiz, Irene, Fernandez, Ovidio, Caballero-Diaz, Cristina, Mellado, Begona, Marrupe, David, Garcia-Sanchez, Jose, Sanchez-Escribano, Ricardo, Fernandez Parra, Eva, Villa Guzman, Jose C., Martinez-Ortega, Esther, Belen Gonzalez, Maria, Moran, Marina, Suarez-Paniagua, Beatriz, Castellano, Daniel. Impact on clinical practice of the implementation of guidelines for the toxicity management of targeted therapies in kidney cancer. The protect-2 study. BMC Cancer. 2016. 16:135

<u>Does not involve a study design of interest(reviews, case reports, letters, comments, protocols, guidelines, single arm studies) (n=15)</u>

41. Saha, S., Mallick, I., Chatterjee, S.. Adjuvant Radiation Therapy Nodal Target Volumes in Oral Cancer: Do Consensus Guidelines Reflect Patterns of Failure?. International Journal of Radiation Oncology, Biology, Physics. 2021. 111:e393-e394
42. Gordian-Arroyo, Adlin M., Zynger, Debra L., Tozbikian, Gary H.. Impact of the 2018 ASCO/CAP HER2 Guideline Focused Update. American Journal of Clinical Pathology. 2019. 152:17-26
43. Guttman Krader, Cheryl. Bladder Ca guideline adherence boosts survival: Significant variation in receipt of guideline-based care observed, however. Urology Times. 2018. 46:11-17
44. Luckett, Tim, Phillips, Jane, Agar, Meera, Lam, Lawrence, Davidson, Patricia M., McCaffrey, Nicola, Boyle, Frances, Shaw, Tim, Currow, David C., Read, Alison, Hosie, Annmarie, Lovell, Melanie. Protocol for a phase III pragmatic stepped wedge cluster randomised controlled trial comparing the effectiveness and cost-effectiveness of screening and guidelines with, versus without, implementation strategies for improving pain in adults with cancer attending outpatient oncology and palliative care services: the Stop Cancer PAIN trial. BMC Health Services Research. 2018. 18:N.PAG-N.PAG
45. Sandler, Kiri A., Shaverdian, Narek, Cook, Ryan R., Kishan, Amar U., King, Christopher R., Yang, Isaac, Steinberg, Michael L., Lee, Percy. Treatment trends for patients with brain metastases: Does practice reflect the data?. Cancer (0008543X). 2017. 123:2274-2282
46. Tetsusuke, Yoshimoto, Shiro, Tomiyasu, Toshinari, Saeki, Tomohiro, Tamaki, Takahiro, Hashizume, Masahiko, Murakami, Motohiro, Matoba. How Do Hospital Palliative Care

Teams Use the WHO Guidelines to Manage Unrelieved Cancer Pain? A 1-Year, Multicenter Audit in Japan. American Journal of Hospice & Palliative Medicine. 2017. 34:92-99
47. Booth, C. M., Nanji, S., Wei, X., Peng, Y., Biagi, J. J., Hanna, T. P., Krzyzanowska, M. K., Mackillop, W. J.. Adjuvant Chemotherapy for Stage II Colon Cancer: Practice Patterns and Effectiveness in the General Population. Clinical Oncology. 2017. 29:e29-e38
48. Chih-Yang, Hsiao, Ching-Yao, Yang, Jin-Ming, Wu, Ting-Chun, Kuo, Yu-Wen, Tien, Hsiao, Chih-Yang, Yang, Ching-Yao, Wu, Jin-Ming, Kuo, Ting-Chun, Tien, Yu-Wen. Utility of the 2006 Sendai and 2012 Fukuoka guidelines for the management of intraductal papillary mucinous neoplasm of the pancreas: A single-center experience with 138 surgically treated patients. Medicine. 2016. 95:1-6
49. Maresso, Karen Colbert, Hawk, Ernest. Cancer Prevention Recommendations: Impact of Adherence. Seminars in Oncology Nursing. 2016. 32:306-313
50. Burch, Henry B., Burman, Kenneth D., Cooper, David S., Hennessey, James V., Vietor, Nicole O.. A 2015 Survey of Clinical Practice Patterns in the Management of Thyroid Nodules. Journal of Clinical Endocrinology & Metabolism. 2016. 101:2853-2862
51. Zhao, Changqing, Nguyen, Mindie H.. Hepatocellular Carcinoma Screening and Surveillance: Practice Guidelines and Real-Life Practice. Journal of Clinical Gastroenterology. 2016. 50:120-133
52. Heller, Danielle R., Jean, Raymond A., Kurbatov, Vadim, Chiu, Alexander S., Khan, Sajid A.. Practice Patterns and Guideline Non-Adherence in Surgical Management of Appendiceal Carcinoid Tumors. Journal of the American College of Surgeons. 2018. 227:e218-e218
53. Wright, Byron E., Lee, Chris C., Iddings, Douglas M., Kavanagh, Maihgan, Bilchik, Anton J.. Management of T2 gallbladder cancer: are practice patterns consistent with national recommendations?. American Journal of Surgery. 2007. 194:820-826
54. Cai, Ming, Cotter, Robin R, Wong, Sandra L, Mayo, Sara W 2024//. Evaluating clinical practice guidelines for the management of rectal cancer: Did they get it RIGHT?. Journal of surgical oncology, 129(8): 1475
55. Shapiro, Jean A, Holub, Jennifer L, Dominitz, Jason A, Sabatino, Susan A, Nadel, Marion R 2024//. Colonoscopy quality measures and adherence to follow-up guidelines among endoscopists participating in a U.S. endoscopy registry.

APPENDIX D: Additional figures and sensitivity analysis

Figure 1: Recurrence free survival

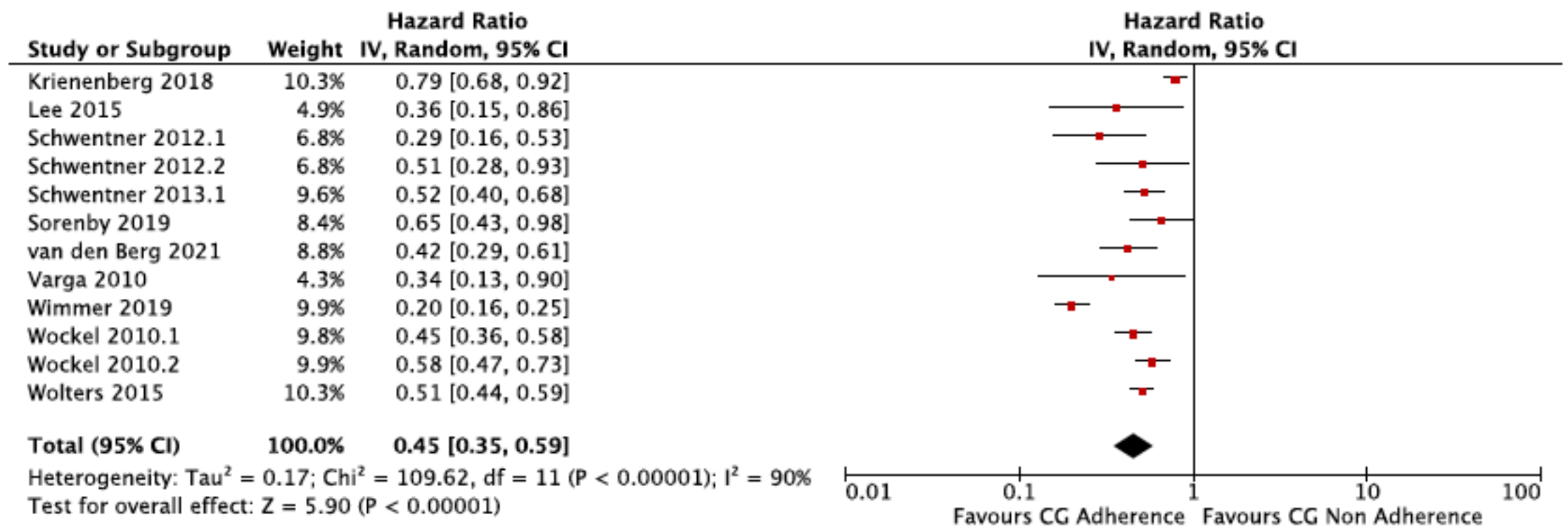

Figure 2: Disease free survival

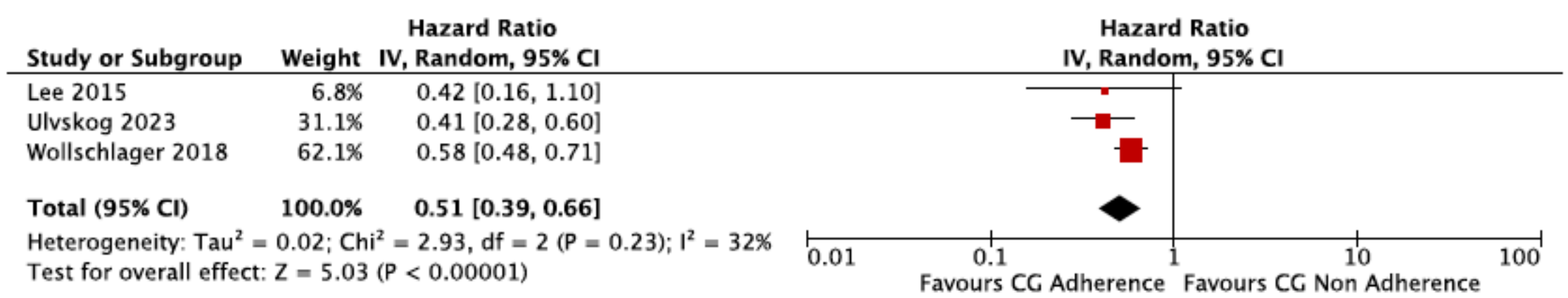

Figure 3: Funnel plot of all studies in meta-analysis

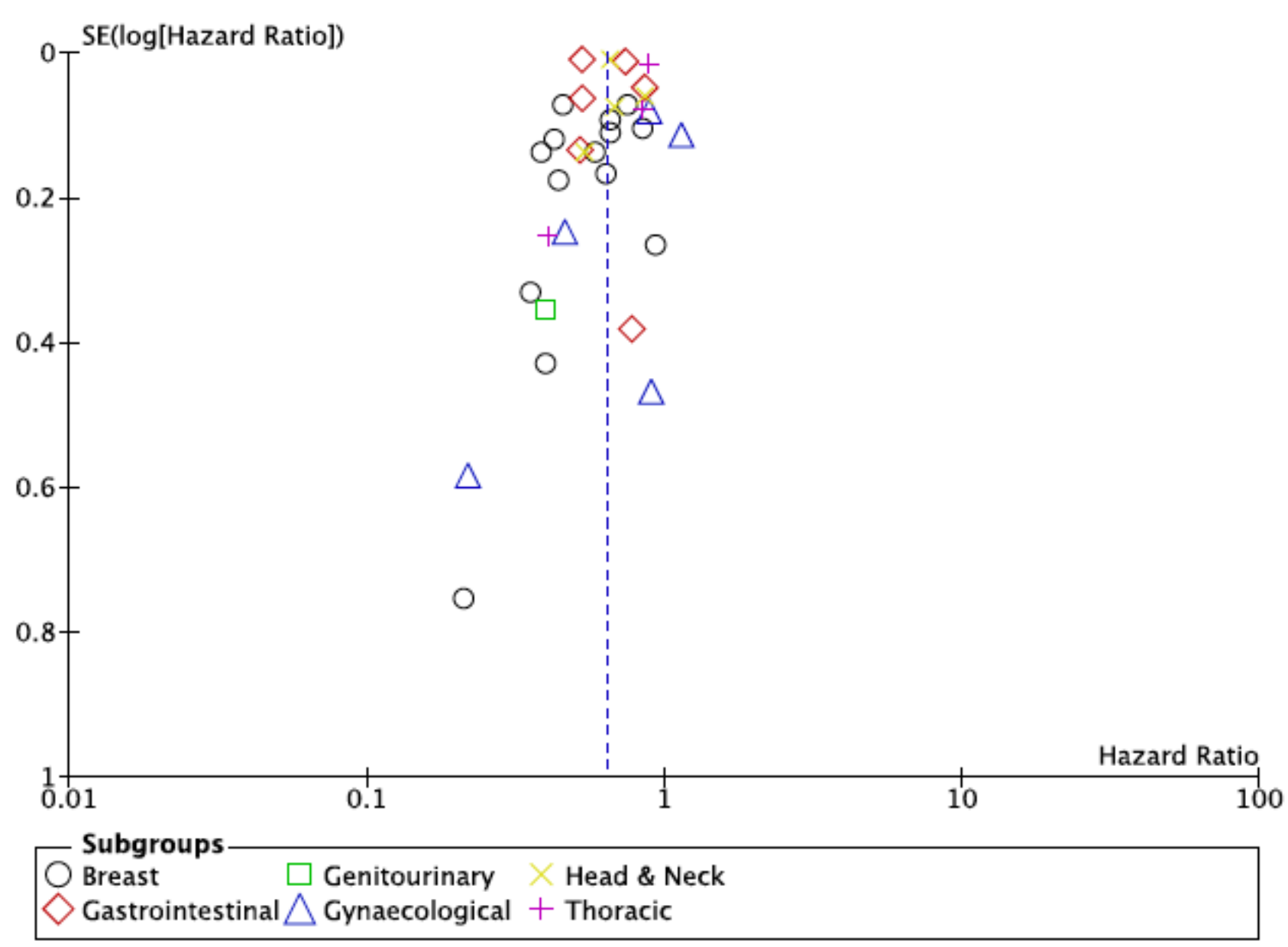

Table 2: Leave one out Meta-analysis

Study Excluded	Disease Site Pooled Effect Size			Overall Pooled Effect Size		
	HR [95% CI]	P-value;	I^2(%)	HR [95% CI]	P-value	I^2(%)
**All Breast**	**0.57 [0.48, 0.67]**	**<0.0001**	**80**	**0.64 [0.59, 0.70]**	**<0.0001**	**97**
Andreano 2017	0.56 [0.47, 0.66]	<0.0001	81	0.64 [0.59, 0.70]	<0.0001	97
Krienerberg 2018	0.55 [0.47, 0.65]	<0.0001	76	0.64 [0.58, 0.70]	<0.0001	97
Rocque 2018	0.55 [0.46, 0.64]	<0.0001	76	0.63 [0.58, 0.69]	<0.0001	97
Sacerdote 2013	0.55 [0.47, 0.65]	<0.0001	80	0.64 [0.58, 0.70]	<0.0001	97
Schwentner 2012.1	0.58 [0.49, 0.68]	<0.0001	81	0.65 [0.59, 0.71]	<0.0001	97
Schwentner 2012.2	0.57 [0.49, 0.67]	<0.0001	81	0.64 [0.59, 0.70]	<0.0001	97
Schwentner 2013.1	0.58 [0.49, 0.68]	<0.0001	80	0.65 [0.59, 0.71]	<0.0001	97
Song 2022	0.56 [0.47, 0.67]	<0.0001	81	0.64 [0.59, 0.70]	<0.0001	97
Varga 2010	0.57 [0.49, 0.67]	<0.0001	81	0.64 [0.59, 0.70]	<0.0001	97
Wimmer 2019	0.56 [0.47, 0.66]	<0.0001	81	0.64 [0.59, 0.70]	<0.0001	97
Wockel 2010.1	0.59 [0.50, 0.69]	<0.0001	78	0.65 [0.60, 0.71]	<0.0001	97
Wockel 2010.2	0.58 [0.49, 0.69]	<0.0001	79	0.65 [0.60, 0.71]	<0.0001	97
Wollschlager 2018	0.56 [0.47, 0.66]	<0.0001	81	0.64 [0.59, 0.70]	<0.0001	97
Wolters 2015	0.58 [0.49, 0.68]	<0.0001	75	0.65 [0.60, 0.71]	<0.0001	97
**All Gastrointestinal**	**0.64 [0.52, 0.78]**	**<0.0001**	**99**	**0.64 [0.59, 0.70]**	**<0.0001**	**97**
Bagante 2018	0.61 [0.48, 0.79]	0.0001	96	0.63 [0.58, 0.70]	<0.0001	97
Guarino 2015	0.63 [0.51, 0.78]	<0.0001	99	0.64 [0.59, 0.70]	<0.0001	97
Jaap 2018	0.67 [0.56, 0.80]	<0.0001	90	0.66 [0.61, 0.71]	<0.0001	93
Kumar 2023	0.66 [0.52, 0.84]	0.0006	99	0.65 [0.59, 0.71]	<0.0001	97
Munir 2023	0.59 [0.47, 0.74]	<0.0001	99	0.63 [0.58, 0.69]	<0.0001	97
Zhao 2018	0.66 [0.53, 0.83]	0.0003	99	0.65 [0.59, 0.71]	<0.0001	97
**All Genitourinary**	**0.40 [0.20, 0.80]**	**0.010**	**NA**	**0.64 [0.59, 0.70]**	**<0.0001**	**97**
Cindolo 2019	0.40 [0.20, 0.80]	0.010	NA	0.65 [0.59, 0.70]	<0.0001	97
**All Gynecological**	**0.76 [0.53, 1.08]**	**0.13**	**77**	**0.64 [0.59, 0.70]**	**<0.0001**	**97**
Chiew 2017	0.85 [0.62, 1.16]	0.31	74	0.65 [0.59, 0.70]	<0.0001	97
Howell 2000	0.60 [0.35, 1.04]	0.07	74	0.63 [0.57, 0.69]	<0.0001	97
Jochum 2021	0.89 [0.64, 1.24]	0.50	69	0.65 [0.59, 0.71]	<0.0001	97
Lankveld 2006	0.73 [0.49, 1.09]	0.12	83	0.64 [0.59, 0.70]	<0.0001	97
Shigeta 2017	0.63 [0.32, 1.25]	0.19	83	0.63 [0.58, 0.69]	<0.0001	97
**All Head and Neck**	**0.69 [0.60, 0.81]**	**<0.0001**	**85**	**0.64 [0.59, 0.70]**	**<0.0001**	**97**
Adam 2015	0.66 [0.63, 0.70]	<0.0001	14	0.63 [0.58, 0.69]	<0.0001	97
Cohen 2022	0.70 [0.55, 0.89]	0.003	83	0.63 [0.57, 0.70]	<0.0001	97
Frisco 2023	0.73 [0.62, 0.86]	0.0002	89	0.65 [0.59, 0.71]	<0.0001	97
Schwam 2016	0.69 [0.56, 0.86]	0.0007	90	0.64 [0.58, 0.70]	<0.0001	97
**All Thoracic**	**0.79 [0.64, 0.97]**	**0.02**	**80**	**0.64 [0.59, 0.70]**	**<0.0001**	**97**
Duggan 2016	0.89 [0.86, 0.92]	<0.0001	0	0.65 [0.59, 0.71]	<0.0001	97
John 2021	0.61 [0.30, 1.23]	0.17	86	0.64 [0.59, 0.69]	<0.0001	95
Yue 2014	0.63 [0.30, 1.34]	0.23	89	0.63 [0.58, 0.69]	<0.0001	97

Figure 4: Subgroup analysis by study duration

Study or Subgroup	Weight	Hazard Ratio IV, Random, 95% CI
**1.4.1 0 to <5 years**		
Chiew 2017	0.5%	0.22 [0.07, 0.69]
Cindolo 2019	1.2%	0.40 [0.20, 0.80]
Guarino 2015	1.1%	0.78 [0.37, 1.64]
Jochum 2021	1.9%	0.47 [0.29, 0.76]
John 2021	4.6%	0.89 [0.86, 0.92]
Krienenberg 2018	4.1%	0.75 [0.65, 0.87]
Kumar 2023	4.2%	0.53 [0.47, 0.61]
Munir 2023	4.4%	0.86 [0.78, 0.95]
Rocque 2018	3.7%	0.85 [0.69, 1.05]
Sacerdote 2013	1.8%	0.94 [0.56, 1.58]
Schwentner 2012.1	1.3%	0.36 [0.19, 0.68]
Schwentner 2012.2	0.9%	0.40 [0.17, 0.92]
Wockel 2010.1	3.2%	0.39 [0.30, 0.51]
Zhao 2018	3.3%	0.52 [0.40, 0.68]
**Subtotal (95% CI)**	**36.2%**	**0.62 [0.52, 0.73]**
Heterogeneity: $Tau^2 = 0.06$; $Chi^2 = 132.20$, df = 13 ($P < 0.00001$); $I^2 = 90\%$		
Test for overall effect: $Z = 5.58$ ($P < 0.00001$)		
**1.4.2 5 to <10 years**		
Adam 2015	4.3%	0.86 [0.76, 0.97]
Andreano 2017	3.9%	0.66 [0.55, 0.79]
Bagante 2018	4.6%	0.74 [0.72, 0.76]
Cohen 2022	4.6%	0.66 [0.65, 0.68]
Duggan 2016	1.9%	0.41 [0.25, 0.67]
Frisco 2023	3.2%	0.54 [0.41, 0.71]
Howell 2000	3.5%	1.15 [0.92, 1.44]
Schwam 2016	4.1%	0.68 [0.59, 0.79]
Shigeta 2017	4.0%	0.89 [0.76, 1.04]
Song 2022	3.2%	0.59 [0.45, 0.78]
Varga 2010	0.3%	0.21 [0.05, 0.93]
Yue 2014	4.1%	0.84 [0.72, 0.98]
**Subtotal (95% CI)**	**41.7%**	**0.74 [0.68, 0.80]**
Heterogeneity: $Tau^2 = 0.01$; $Chi^2 = 101.62$, df = 11 ($P < 0.00001$); $I^2 = 89\%$		
Test for overall effect: $Z = 7.46$ ($P < 0.00001$)		
**1.4.3 10 years and above**		
Jaap 2018	4.6%	0.53 [0.52, 0.54]
Lankveld 2006	0.8%	0.90 [0.36, 2.25]
Schwentner 2013.1	2.7%	0.44 [0.31, 0.63]
Wimmer 2019	2.8%	0.64 [0.46, 0.89]
Wockel 2010.2	3.5%	0.43 [0.34, 0.54]
Wollschlager 2018	3.6%	0.66 [0.53, 0.82]
Wolters 2015	4.1%	0.46 [0.40, 0.53]
**Subtotal (95% CI)**	**22.1%**	**0.52 [0.47, 0.58]**
Heterogeneity: $Tau^2 = 0.01$; $Chi^2 = 14.33$, df = 6 ($P = 0.03$); $I^2 = 58\%$		
Test for overall effect: $Z = 11.33$ ($P < 0.00001$)		
**Total (95% CI)**	**100.0%**	**0.64 [0.59, 0.70]**
Heterogeneity: $Tau^2 = 0.04$; $Chi^2 = 1055.45$, df = 32 ($P < 0.00001$); $I^2 = 97\%$		
Test for overall effect: $Z = 9.91$ ($P < 0.00001$)		
Test for subgroup differences: $Chi^2 = 24.75$, df = 2 ($P < 0.00001$), $I^2 = 91.9\%$		

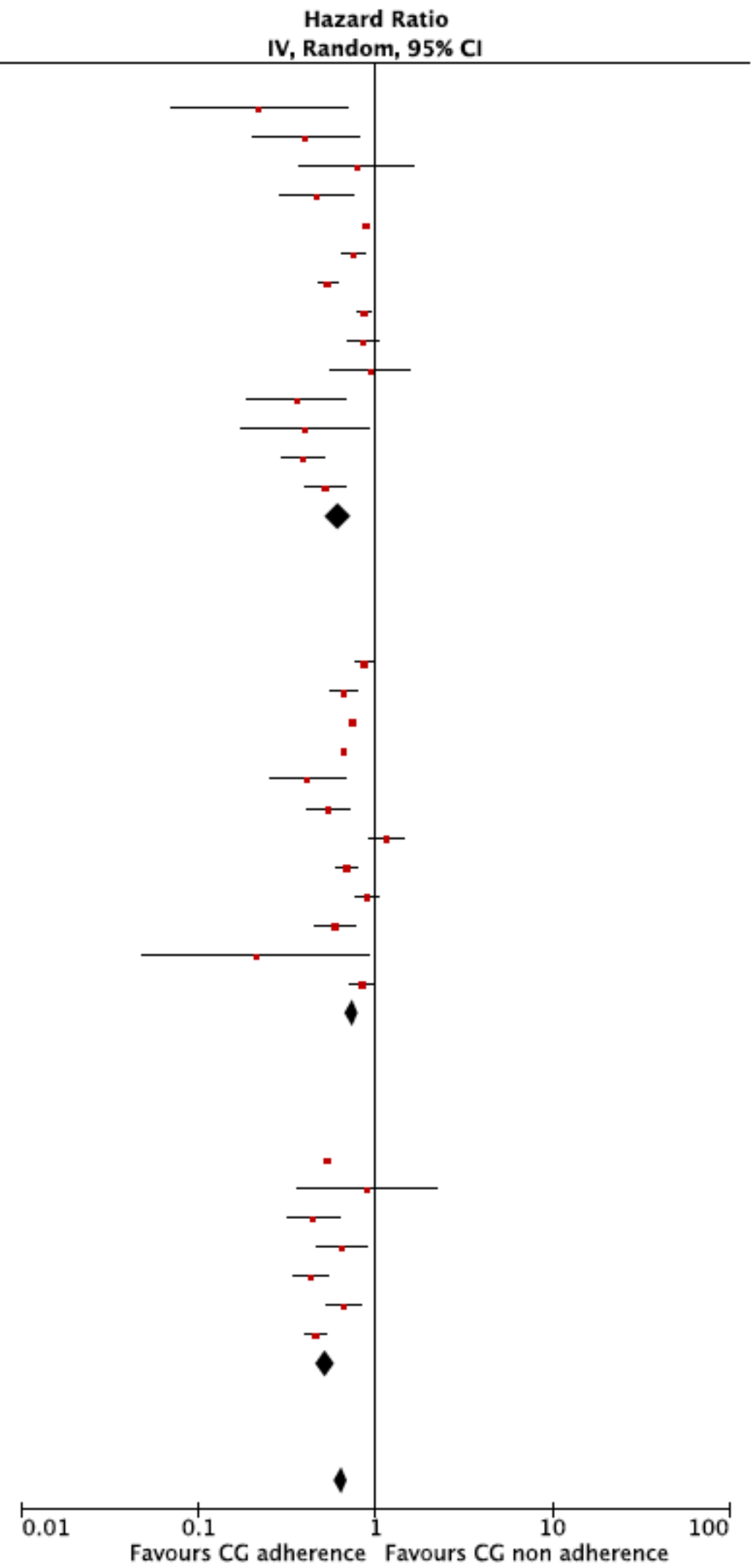

Figure 5: Summary of subgroup analysis

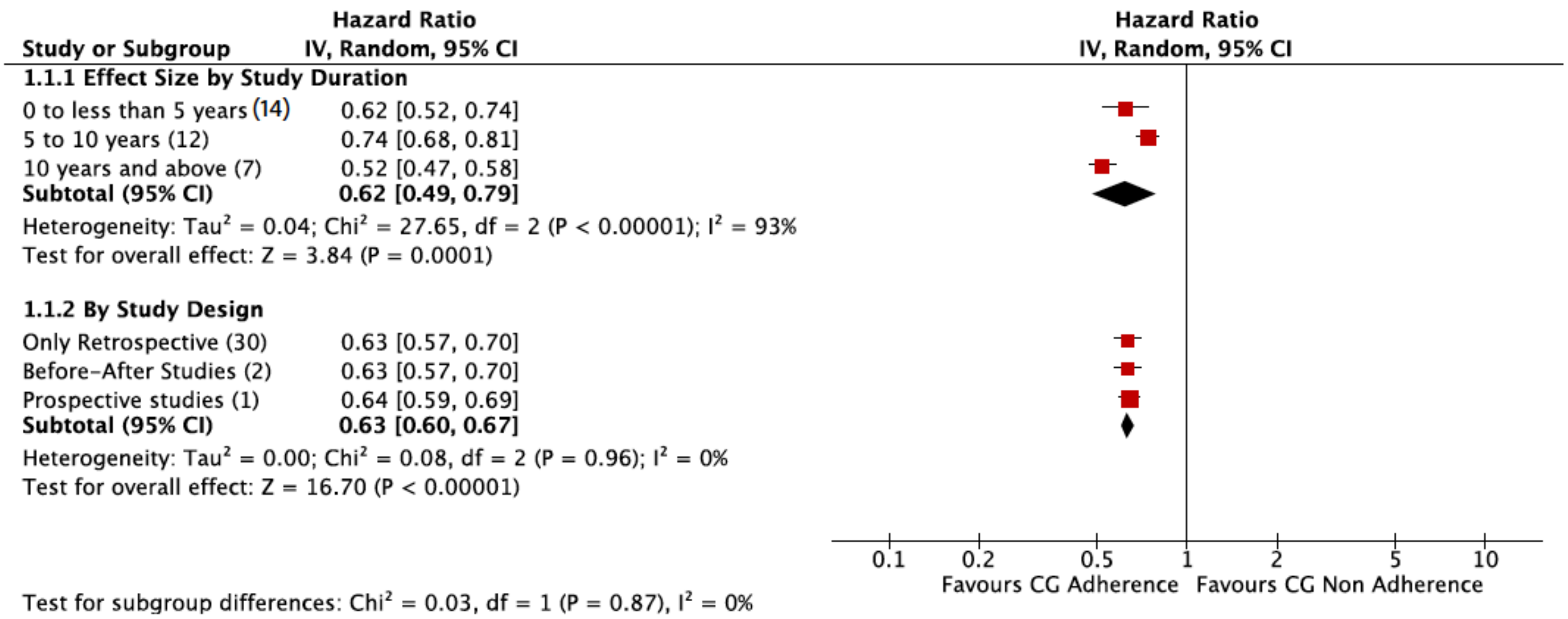

Figure 6: Leave one out sensitivity meta-analysis.

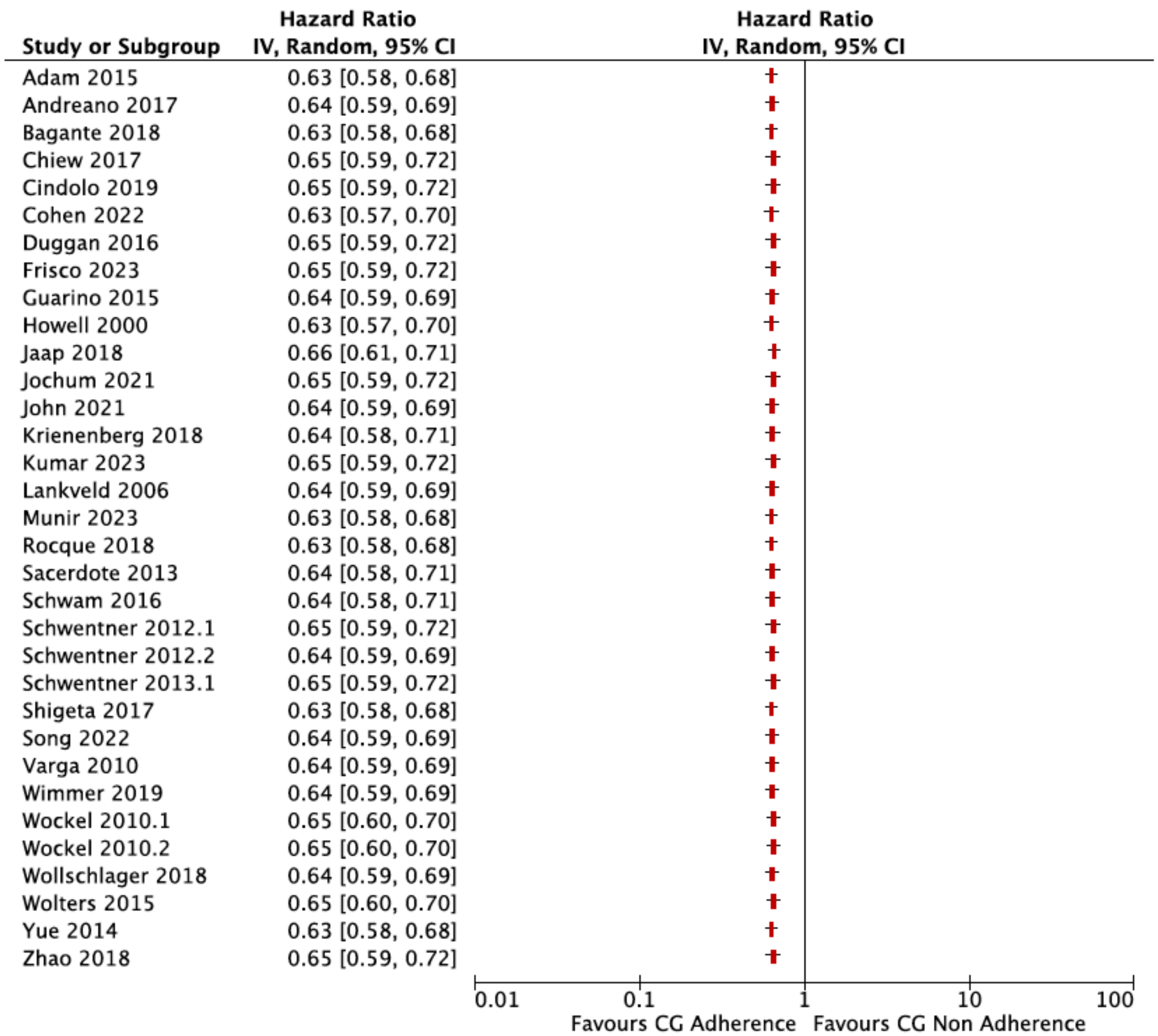

Supplement: Supplementary file 1 [file GIN2-2-e70030-s001.pdf]
